# The Vertebral Artery: A Systematic Review and a Meta-Analysis of the Current Literature

**DOI:** 10.3390/diagnostics13122036

**Published:** 2023-06-12

**Authors:** Răzvan Costin Tudose, Mugurel Constantin Rusu, Sorin Hostiuc

**Affiliations:** 1 Division of Anatomy, Faculty of Dentistry, “Carol Davila” University of Medicine and Pharmacy, 020021 Bucharest, Romania; razvan-costin.tudose0721@stud.umfcd.ro; 2 Division of Legal Medicine and Bioethics, Faculty of Dentistry, “Carol Davila” University of Medicine and Pharmacy, 050474 Bucharest, Romania; sorin.hostiuc@umfcd.ro

**Keywords:** neck arteries, computed tomography, origin, tortuosity, hypoplasia

## Abstract

(1) Background. The anatomical variations of the vertebral arteries (VAs) have a significant impact both in neurosurgery and forensic pathology. The purpose of this study was to evaluate the variational anatomy of the vertebral artery. We evaluated anatomical aspects regarding the V1 and V2 segments of the VA: origin, course, tortuosity, hypoplasia, and dominance, and established the prevalence of each variation. (2) Methods. We conducted a systematic search in PubMed and Google Scholar databases, up to December 2022. Sixty-two studies, comprising 32,153 vessels, were included in the current meta-analysis. We used a random-effects model with a DerSimonian-Laird estimator. The confidence intervals were set at 95%. The heterogeneity between studies was assessed using I^2^. The funnel plot and Egger’s regression test for plot asymmetry were used for the evaluation of publication bias. Statistical significance was considered at *p* < 0.05. (3) Results. The most common site for the origin of both VAs was the subclavian artery. The aortic arch origin of the left VA had a prevalence of 4.81%. Other origins of the right VAs were noted: aortic arch (0.1%), right common carotid artery (0.1%), and brachiocephalic trunk (0.5%). Ninety-two percent of the VAs entered the transverse foramen (TF) of the C6 vertebra, followed by C5, C7, C4, and least frequently, C3 (0.1%). Roughly one out of four (25.9%) VAs presented a sort of tortuosity, the transversal one representing the most common variant. Hypoplasia occurred in 7.94% of the vessels. Left VA dominance (36.1%) is more common, compared to right VA dominance (25.3%). (4) Conclusions. The anatomy of the VA is highly irregular, and eventual intraoperative complications may be life-threatening. The prevalence of VA origin from the subclavian artery is 94.1%, 92.0% of the VAs entered the TF at C6, 26.6% were tortuous, and 7.94% were hypoplastic.

## 1. Introduction

The vertebral arteries (VA), left (LVA) and right (RVA), supply the upper spinal cord, brainstem, cerebellum, and the posterior part of the brain with oxygenated blood, accounting for 28% of its supply [1,2]. Commonly, the VA originates from the first cervical part of the subclavian artery (SA), medial to the anterior scalene muscle, continuing its ascending course through the transverse foramina (TF) of cervical transverse processes, passing through the foramen magnum, to end in the posterior fossa by forming the basilar artery with the opposite VA [3].

**Figure 1:**
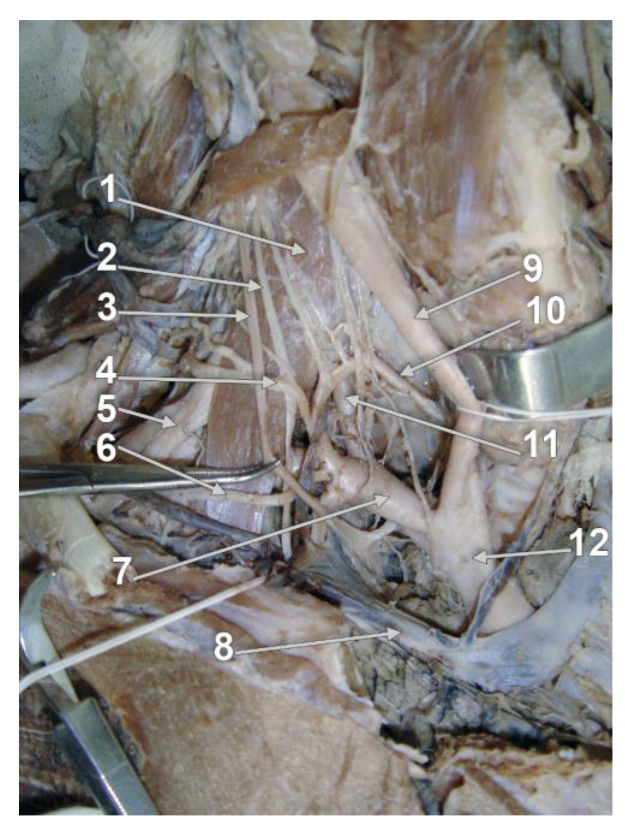
Dissection of the V1 segment of the right vertebral artery in the scalenovertebral triangle. Major veins, the right clavicle, the first rib and, partly, the manubrium, were removed: 1. anterior scalene muscle; 2. phrenic nerve; 3. vagus nerve; 4. transverse cervical artery; 5. brachial plexus; 6. suprascapular artery; 7. subclavian artery; 8. right brachiocephalic vein; 9. common carotid artery; 10. inferior thyroid artery; 11. V1 segment of vertebral artery; 12. brachiocephalic trunk.

For an improved overview of the VA, it has been divided anatomically into four segments, three extracranial, and one intracranial [4,5]. The first V1 segment (extraosseous, prevertebral or pretransverse, ostial, proximal) describes the course of the VA from its origin until it enters the TF of (usually) either the fifth or the sixth cervical vertebra (Figure 1) [6,7]. The second V2 segment (cervical, foraminal, transforaminal), ascends through the TF of C6 to C2 [8], continuing with the third V3 segment (atlantal/atlantic [9,10], extraspinal) behind the lateral mass of the atlas, further entering the foramen magnum. The final intracranial and intradural V4 segment starts as the VA perforates the dura and arachnoid mater in the foramen magnum and pursues its ascending course on clivus. At the lower border of the pons, the confluence of the VAs forms the basilar artery [11].

The study aimed to assess the morphology of the VA’s V1 and V2 segments from a qualitative perspective. There were evaluated the course of the VA, the origin, the vertebral level of entrance in the TF, tortuosity and straightness, planes of tortuosity, hypoplasia, and dominance of this artery.

## 2. Materials and Methods

The study was performed according to PRISMA (Preferred Reporting Items for Systematic Reviews and Meta-Analyses) guidelines for reporting systematic reviews and meta-analyses [12].

### 2.1. Search Method

Articles were obtained by performing systematic searches on different databases, PubMed and Web of Science, using the following keywords: “Vertebral artery” AND “Anatomy” AND “V1” and “Vertebral artery” AND “Anatomy” AND “V2”. The reference list of all the relevant articles was thoroughly scrutinized for other appropriate references to be included in the analysis. Extensive searches were also performed on Google Scholar, Google, and Research Gate. Every potentially relevant article was obtained as a full article, analyzed by two reviewers, and included in the current paper if it fulfilled the inclusion criteria and did not fulfill any of the exclusion criteria. Selected articles were imported into a Paperpile database.

### 2.2. Selection Criteria

The following exclusion criteria were used: (1) articles that did not mention any relevant measurements regarding the first two segments of the VA, (2) results duplicating previously published articles, (3) low-quality articles comprising inadequate methods of measurement and irrelevant results or published in unknown/low impact factor journals, (4) less than 10 subjects, (5) studies conducted on fetuses, (6) inability to obtain the full article, (7) studies that measured the prevalence of tortuosity in patients with Loeys–Dietz syndrome, Marfan syndrome or any other vascular disease, as the frequency of tortuous vessels in these subjects is known to be much higher compared to healthy subjects [13,14], (8) case reports, reviews, meta-analyses, or any other prevalence studies based on published values.

### 2.3. Data Collection and Analysis

Two reviewers separately extracted data from each study in different Excel 365 databases. If any major discrepancy appeared, a third reviewer was co-opted to check the inconsistency and select the appropriate result. The following information was extracted: name of the first author, year, method (CT, autopsy reports, surgical reports, etc.), total number of cases, total number of left and right VAs (LVAs and RVAs), the analyzed segment of the VA (V1 or V2) and its measurements, each in a separate, specific column.

### 2.4. Quality Assessment and Risk of Bias

Newcastle–Ottawa Scale (NOS) for case-control studies was used, with the following adjustments for a prevalence analysis: item 3 from Selection and Exposure was excluded. Each article was noted with a mark ranging from 0 to 7 to assess the quality. Articles with at least 4 points were included in the current paper.

### 2.5. Statistical Analysis

Results were statistically analyzed using Jamovi 2.3 software (Sydney, Australia) for data analysis. Three columns were created: authors, total cases, and the third one which varied, depending on the measured variable. The “Proportions” tab was used to process the variables. The “Total cases” column also varied in accordance with the variable. If the analyzed variable concerned only one side (left or right), then the standardized/true prevalence for that particular side was determined. The following analyses were conducted by determining the crude prevalence for each anatomical variant: C3, C4, C5, C6, and C7 TF entrance, overall tortuosity, VA dominance, and hypoplasia. VA origin, TF entrance corelated to VA origin and plane of tortuosity were assessed as standardized prevalence, as well as the results reported in Table 1, based on study type (autopsy or imaging). Cases that did not mention the exact number of VAs (e.g., 70 specimens) were analyzed carefully. If, after scrutinizing the entire article, the published data indicated a number of arteries double the mentioned one, we considered the first as the real number of VAs (70 LVAs and 70 RVAs) and the latter as the number of patients [15]. The following configuration was used for the statistical processing of the data. The model estimator was DerSimonian–Laird, Raw Proportion was used for effect size model measures, Moderator Type was set on no moderator, and the Confidence Interval (CI) level was established at 95%. Statistical significance was considered at *p* < 0.05. The funnel plot and Egger’s regression test for plot asymmetry were used for the analysis of the publication bias. I^2^ was used to test the presence of heterogeneity between studies, using the following thresholds: 0–35%—most likely not important, 36–55%—moderate heterogeneity, 56–85%—most likely substantial heterogeneity, and 86–100%—significant heterogeneity (average values based on [16]). Considering VA course prevalence, Trattnig et al., (1993) [17] report two types of results, measured on two different groups of subjects (140 and 160 subjects, respectively) and with different methods (cadavers and imagistic). The article was, therefore, used twice in the analyses. Regarding hypoplasia, as each author defines a hypoplastic VA differently, we adopted the highest reported value, to include all the reported measurements. Thus, hypoplasia is interpreted as a diameter of 3.5 mm or less. If reported data of hypoplasia or dominance were split into V1 and V2 segments, they were summed up, so the prevalence of hypoplastic/dominant VA was calculated as a whole for the mentioned segments. Vaiman et al. [18] analyzed a total of 400 VAs, with 23 anomalous VAs. We used 23 as the total number of vessels to determine the prevalences, since the article reported data about the origin and TF entrance only for these anomalous VAs. For each description of the forest plot in the Result section, we reported the number of studies, the total number of vessels included in the analysis, and the number of vessels displaying the anatomical variant. Then, we added the results of the statistical analysis: prevalence, CI, *p*-value, heterogeneity, and Egger’s regression test for plot asymmetry. We also evaluated the standardized prevalence disparity between autopsy and imaging studies and specified whether it was significant or not.

## 3. Results

### 3.1. Search Synthesis

**Figure 2:**
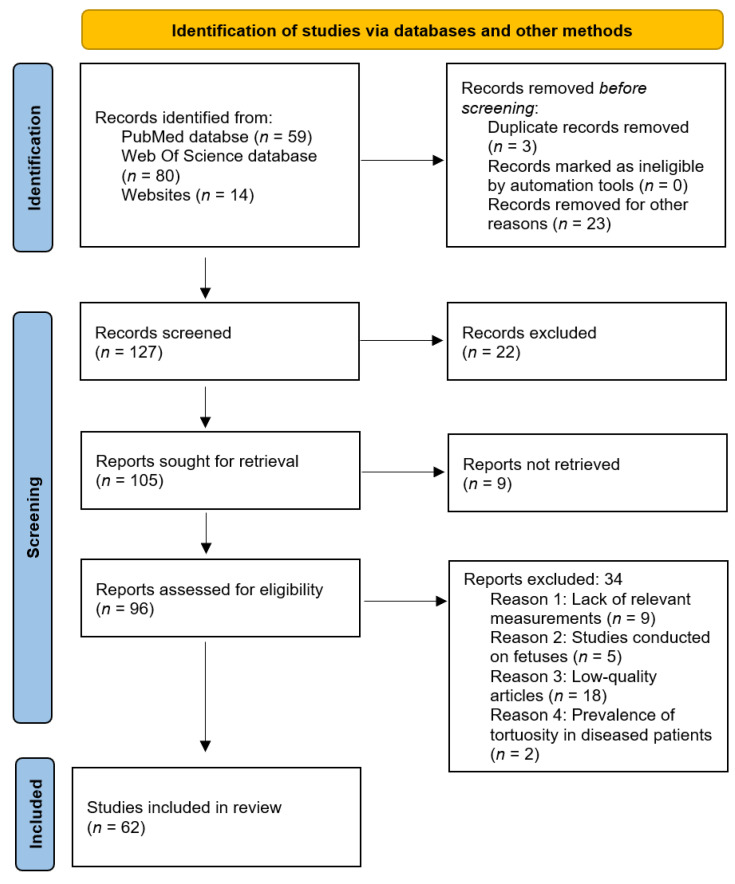
PRISMA flow diagram, adapted after [12].

During the initial research via databases and other methods, 60 references were gathered for V1 segment and 93 for V2. After removing all articles that displayed at least one exclusion criterion, 62 papers were further scrutinized and included in the current meta-analysis. The search synthesis is systemized in Figure 2. Each paper contained in the present study is detailed in Table 2.

### 3.2. Quality Assessment and Risk of Bias

Each article was assessed with a score between 0 and 7. The score for each study was noted in Table 2. There was no significant bias in any of the mentioned papers.

### 3.3. Prevalence of VA Origin

#### 3.3.1. LVA Originating from the SA

**Figure 3:**
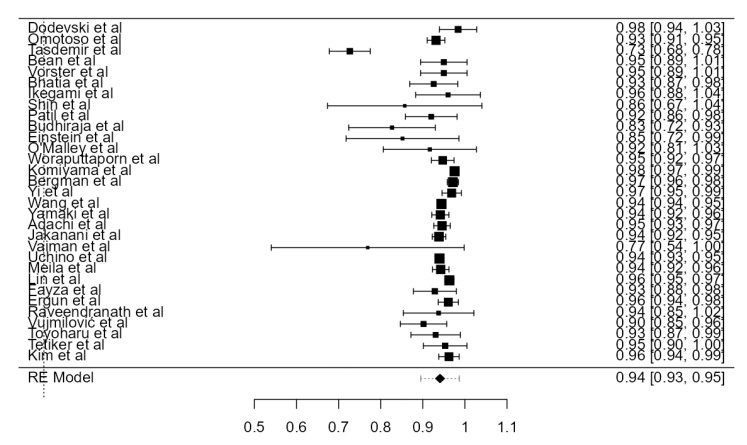
Forest plot—LVA originating from the SA.

Thirty-one studies, adding up to 12,456 LVAs, contained data about the origin of the LVA. The standardized prevalence of the LVA arising from the SA was 94.1% (CI: 93.0–95.2, *p* < 0.001), as shown in Figure 3. The heterogeneity was substantial (I^2^ = 80.7%). Based on Egger’s regression test for plot asymmetry, there was a significant publication bias (*p* < 0.001). No major difference was found between standardized prevalence determined in autopsy (94.5%) and imaging (93.5%) studies (Table 1).

#### 3.3.2. LVA Originating from the Aortic Arch

**Figure 4:**
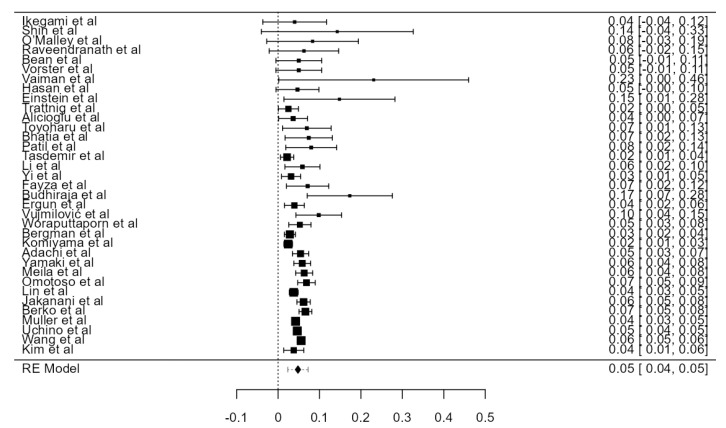
Forest plot—LVA originating from the AA.

LVA arising from the aortic arch (AA) was found in thirty-five original papers, summing up 15,848 assayed LVAs. The prevalence of such anatomical variant was 4.81% (CI: 4.2–5.4, *p* < 0.001), as illustrated in Figure 4. The heterogeneity was substantial (I^2^ = 59.41%). Considering Egger’s regression test for plot asymmetry, there is a significant publication bias (*p* < 0.001). We did not identify any considerable difference between the prevalence determined in autopsy (5.47%) and imaging (4.59%) studies (Table 1).

#### 3.3.3. RVA Originating from the SA

**Figure 5:**
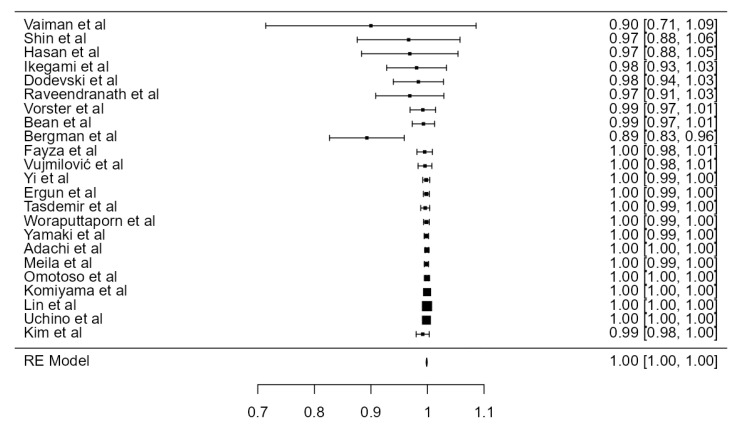
Forest plot—RVA originating from the SA.

The origin of the RVA from the SA (normal or aberrant) was inspected in twenty-three studies. The total amount of investigated RVAs was 8002. The determined prevalence was 99.9% (CI: 99.8–99.9, *p* < 0.001), as shown in Figure 5. The heterogeneity was not important (I^2^ = 0%). Regarding Egger’s plot asymmetry regression test, a significant publication bias was found (*p* < 0.001). No significant prevalence divergence was discovered in autopsy (99.8%) and imaging studies (99.9%) (Table 1).

#### 3.3.4. RVA Originating from the AA

In total, 2992 RVAs were examined in four different original studies that reported the presence of an RVA arising from the AA. This type of origin was only found in 5 RVAs. The prevalence was not statistically significant (*p* = 0.306). The heterogeneity was not important (I^2^ = 11.7%) and the publication bias was not statistically significant (*p* = 0.069), as shown in Table 3.

#### 3.3.5. RVA Originating from the Right Common Carotid Artery

Four articles, analyzing 4368 RVAs, described 8 vessels arising from the right common carotid artery (RCCA). The overall prevalence was 0.126% (CI: 0–0.2, *p* = 0.019), presented in Table 3. The heterogeneity was not important (I^2^ = 0%). Based on Egger’s regression test for plot asymmetry, there was not a significant publication bias (*p* = 0.130).

#### 3.3.6. RVA Originating from the Brachiocephalic Trunk

Three original papers described 9 out of 848 RVAs arising from the brachiocephalic trunk (BCT). The overall prevalence was 0.539% (CI: −0.5–1.6, *p* = 0.325), as shown in Table 3. The heterogeneity was substantial (I^2^ = 73%). Considering Egger’s regression test for plot asymmetry, there is a significant publication bias (*p* = 0.009).

### 3.4. TF Entrance of the VA

#### 3.4.1. C3 TF Entrance Prevalence

##### C3 TF Entrance of the LVAs Originating from the AA

Out of forty LVAs that originated from the AA, four of them entered the TF at the level of the third vertebra. The prevalence was 8.11% (CI: −0.2–16.4, *p* = 0.057), as in Table 3. The heterogeneity was not important (I^2^ = 0%). Based on Egger’s regression test for plot asymmetry, there was not a significant publication bias (*p* = 0.280).

##### C3 TF Entrance

**Figure 6:**
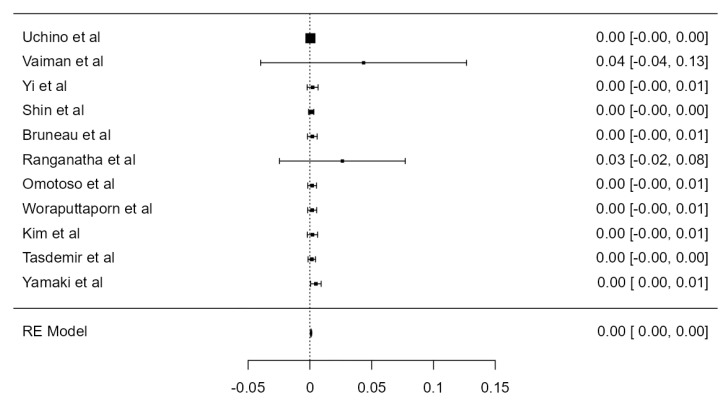
Forest plot—C3 TF entrance.

Considering the crude prevalence of the VAs entering the TF at the level of C3, eleven articles, adding up to 7449 VAs, described 15 vessels presenting such an anatomical variant (Figure 6, Figures S1 and S2). The prevalence (0.1%, CI: 0–0.2) was statistically significant (*p* = 0.008). The heterogeneity was not important (I^2^ = 0%). Publication bias measured with Egger’s regression test for plot asymmetry was significant (*p* = 0.009).

#### 3.4.2. C4 TF Entrance Prevalence

##### C4 TF Entrance of the LVAs Originating from the AA

**Figure 7:**
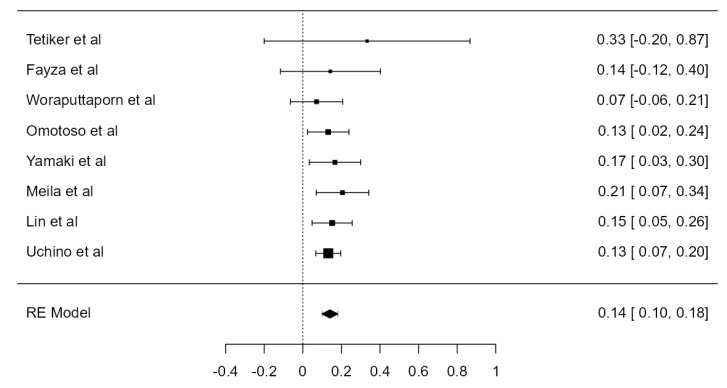
Forest plot—C4 TF entrance of the LVAs originating from the AA.

Eight articles described forty-one out of 278 LVAs arising from the AA that entered the TF at the level of the fourth vertebra. The overall prevalence was 14.1% (CI: 10–18.2, *p* < 0.001), as in Figure 7. The heterogeneity was not important (I^2^ = 0%). Publication bias was not statistically significant (*p* = 0.514).

##### C4 TF Entrance of the LVAs Originating from the SA

Four original papers presented nine out of 2736 LVAs arising from the SA, which entered the TF at C4. The prevalence was 0.337% (CI: −0.2–0.9, *p* = 0.216). The heterogeneity was moderate (I^2^ = 39.11%). The publication bias was statistically significant (*p* = 0.031) (Table 3).

##### C4 TF Entrance of the LVAs

**Figure 8:**
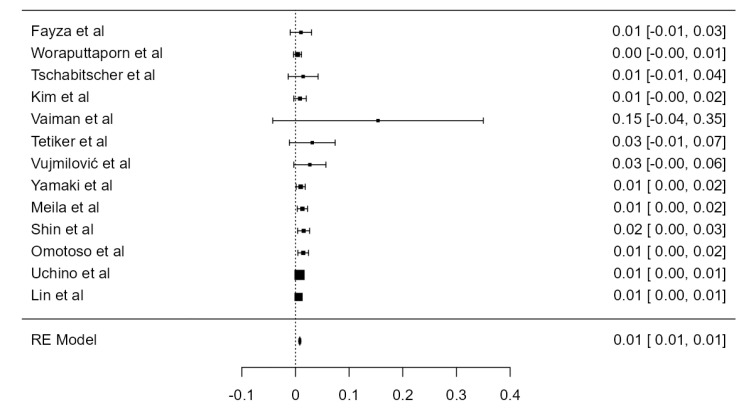
Forest plot—C4 TF entrance of the LVAs.

In total, 6405 LVAs analyzed in thirteen papers described sixty-three which entered the TF at C4. The overall prevalence was 0.807% (CI: 0.6–1.0, *p* < 0.001), as in Figure 8, Figures S3 and S4. The heterogeneity was not important (I^2^ = 0%). Considering Egger’s regression test for plot asymmetry, there is a significant publication bias (*p* = 0.009).

##### C4 TF Entrance of the RVAs

**Figure 9:**
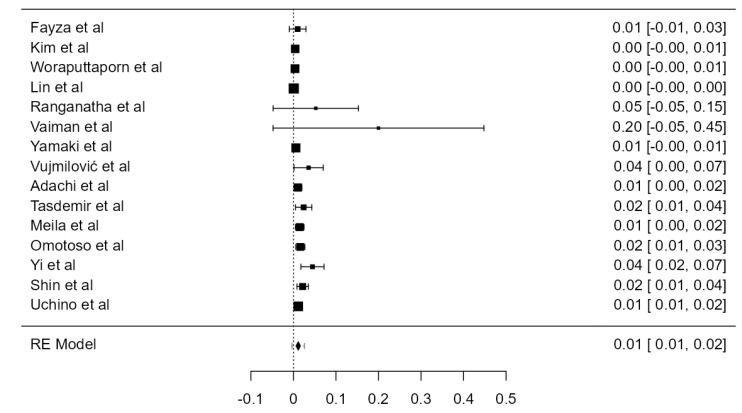
Forest plot—C4 TF entrance of the RVAs.

The TF entrance of 7183 RVAs was analyzed in 15 studies. Eighty-seven were seen entering at the level of the C4 vertebra. The prevalence was 1.14% (CI: 0.6–1.6, *p* < 0.001), shown in Figure 9, Figures S5 and S6. The heterogeneity was substantial (I^2^ = 77.99%). Egger’s regression test for plot asymmetry showed a significant publication bias (*p* < 0.001).

##### C4 TF Entrance

**Figure 10:**
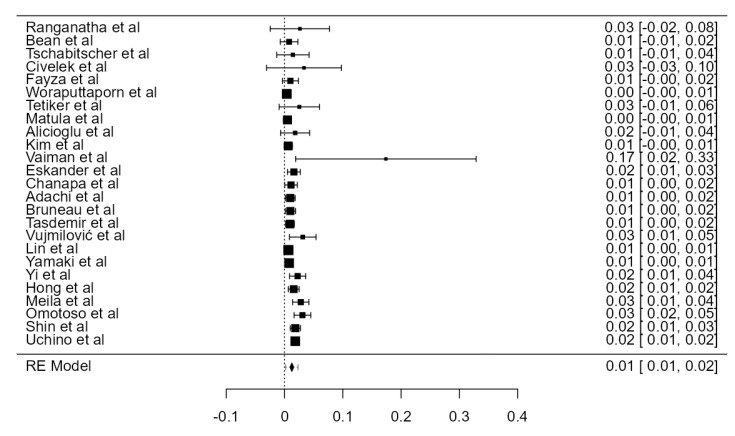
Forest plot—C4 TF entrance.

Twenty-five articles reported the entrance of 184 VAs (out of 12,528 VAs) in the TF of the fourth cervical vertebra. The crude prevalence was estimated at 1.25% (CI: 0.9–1.6, *p* < 0.001), as in Figure 10, Figures S7 and S8. The heterogeneity was moderate (I^2^ = 55.23%). Egger’s regression test for plot asymmetry showed a significant publication bias (*p* < 0.001). A notable disparity was noticed between prevalences from autopsy (0.7%) and imaging (1.45%) studies (Table 1).

#### 3.4.3. C5 TF Entrance Prevalence

##### C5 TF Entrance of the LVAs Originating from the AA

**Figure 11:**
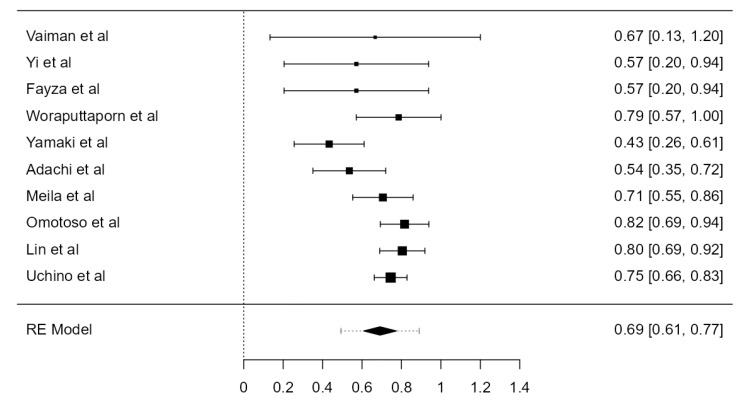
Forest plot—C5 TF entrance of the LVAs originating from the AA.

Ten original papers analyzed 313 LVAs with AA origin. The prevalence of entrance in the TF of the fifth vertebra was 69.2% (CI: 60.8–77.5, *p* < 0.001), represented in Figure 11. The heterogeneity was moderate (I^2^ = 55.69%). The publication bias was not significant (*p* = 0.177).

##### C5 TF Entrance of the LVAs Originating from the SA

**Figure 12:**
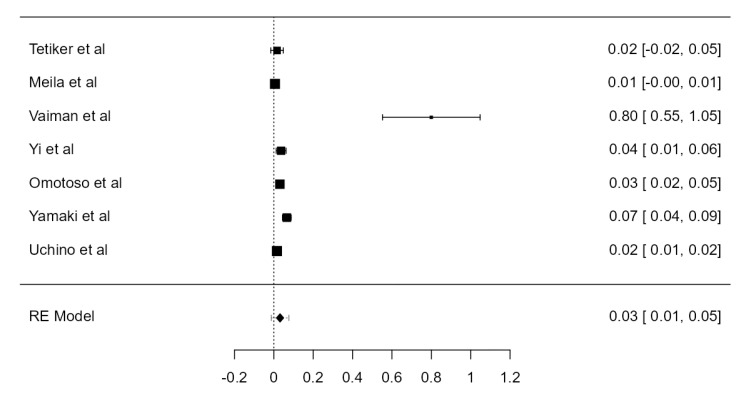
Forest plot—C5 TF entrance of the SA-originating LVAs.

In total, 3944 AA-originated LVAs were scrutinized in seven papers. The prevalence was 3.17% (CI: 1.3–5.0, *p* < 0.001). Both the heterogeneity (I^2^ = 91.83%) and the publication bias (*p* < 0.001) were significant (Figure 12, Figures S9 and S10).

##### C5 TF Entrance of the LVAs

**Figure 13:**
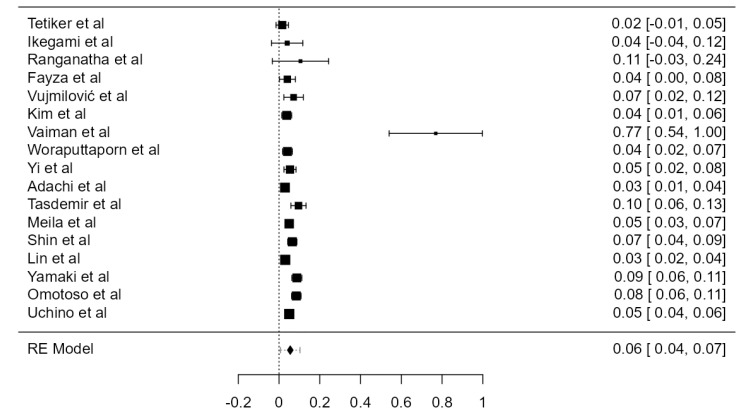
Forest plot—C5 TF entrance of the LVAs.

In total, 7359 LVAs examined in seventeen studies were included in the current evaluation. The prevalence of the TF entrance at C5 was 5.51% (CI: 4.1–6.9, *p* < 0.001), as in Figure 13, Figures S11 and S12. The heterogeneity was substantial (I^2^ = 82.74%). Considering Egger’s regression test for plot asymmetry, there was a significant publication bias (*p* < 0.001).

##### C5 TF Entrance of the RVAs

**Figure 14:**
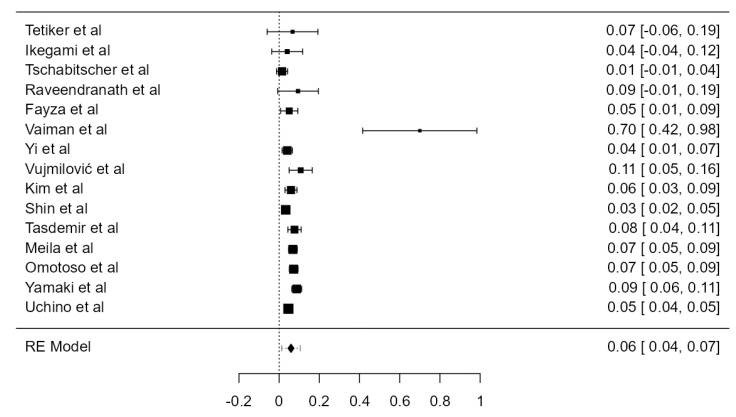
Forest plot—C5 TF entrance of the RVAs.

Analyzing the prevalence of RVAs entering the TF at the level of C5, we found a result of 5.88% (CI: 4.4–7.4, *p* < 0.001). The heterogeneity was substantial (I^2^ = 74.81%), and the publication bias was significant (*p* = 0.002) (Figure 14, Figures S13 and S14).

##### C5 TF Entrance

**Figure 15:**
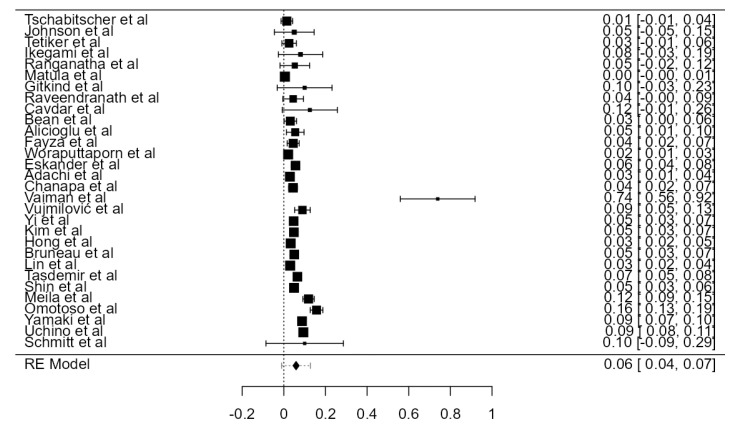
Forest plot—C5 TF entrance.

In total, 12,664 VAs, examined in thirty articles, were included in the present analysis. The crude prevalence of the VA entering the TF at C5 was 5.87% (CI: 4.4–7.3, *p* < 0.001), as shown in Figure 15, Figures S15 and S16. The heterogeneity was significant (I^2^ = 92.73%). Based on Egger’s regression test for plot asymmetry, there was a significant publication bias (*p* < 0.001). We discovered a notable difference between prevalences determined in autopsy (4.37%) and imaging (8.36%) studies (Table 1).

#### 3.4.4. C6 TF Entrance Prevalence

##### C6 TF Entrance of the AA-Originating LVAs

**Figure 16:**
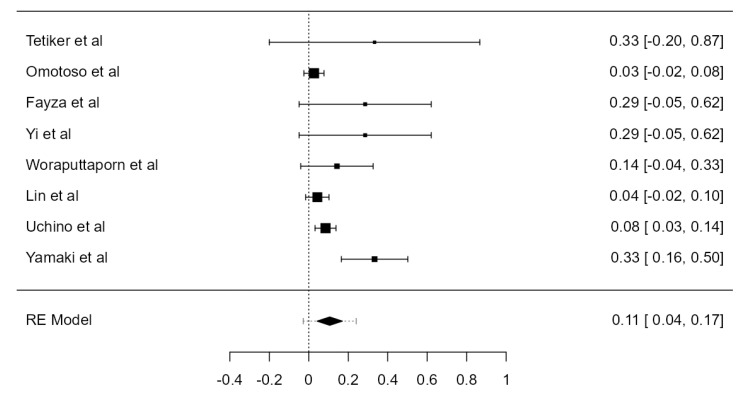
Forest plot—C6 TF entrance of AA-originating LVAs.

In total, 251 LVAs with AA origin from eight papers were evaluated. The prevalence of the C6 TF entrance in such vessels was 10.6% (CI: 4.2–17.0, *p* = 0.001), shown in Figure 16. The heterogeneity was substantial (I^2^ = 61.06%). The publication bias was significant (*p* = 0.001).

##### C6 TF Entrance of the SA-Originated LVAs

**Figure 17:**
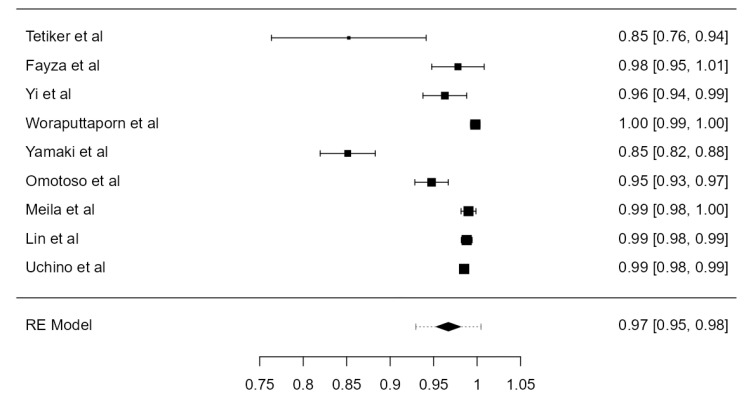
Forest plot—C6 TF entrance of SA-originated LVAs.

In total, 5450 SA-originating VAs from 9 studies were included. The determined prevalence of the C6 TF entrance was 96.7% (CI: 95.3–98.1, *p* < 0.001), presented in Figure 17. The heterogeneity (I^2^ = 93.09%) and the publication bias (*p* < 0.001) were significant.

##### C6 TF Entrance of the LVAs

**Figure 18:**
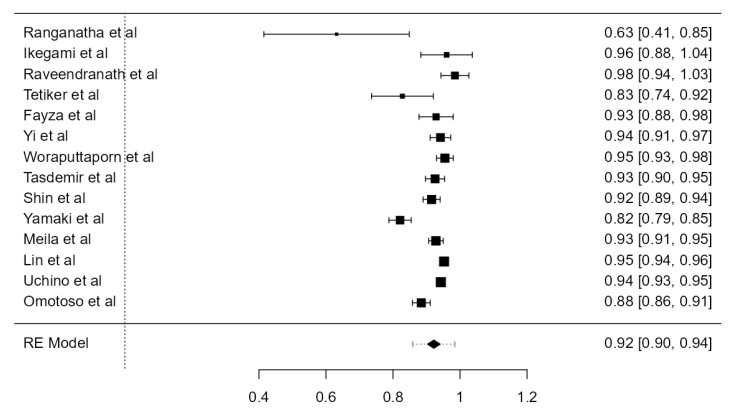
Forest plot—C6 TF entrance of the LVAs.

Fourteen articles, adding up to 6593 LVAs, were included. The overall prevalence was 92.2% (CI: 90.3–94.1, *p* < 0.001), as in Figure 18. Both the heterogeneity (I^2^ = 86.21%) and the publication bias (*p* = 0.014) were significant.

##### C6 TF Entrance of the RVAs

**Figure 19:**
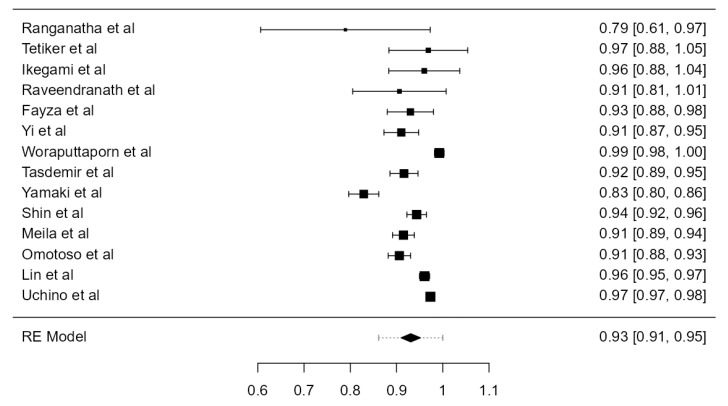
Forest plot—C6 TF entrance of the RVAs.

Fourteen studies, describing 6495 RVAs, contained data about the TF entrance of the RVAs. The overall prevalence was 93.1% (CI: 91.0–95.2, *p* < 0.001), as in Figure 19. The heterogeneity was significant (I^2^ = 92.29%). The publication bias was not significant (*p* = 0.061).

##### C6 TF Entrance

**Figure 20:**
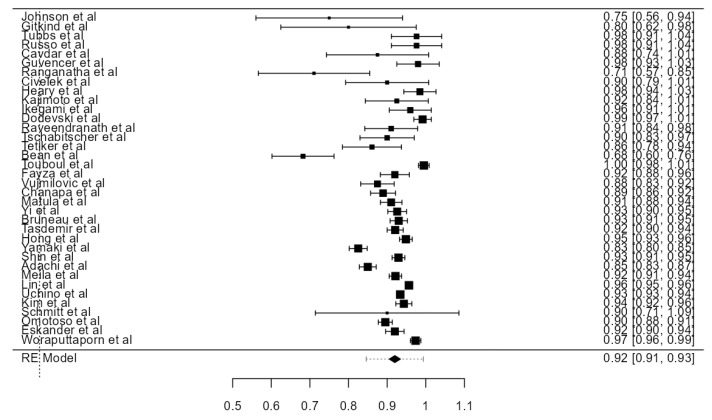
Forest plot—C6 TF entrance.

Thirty-six papers, including 18,002 VAs, were examined and included in the current analysis. The crude prevalence of the VA entering the TF at the C6 level was 92.0% (CI: 90.5–93.4, *p* < 0.001), presented in Figure 20. The heterogeneity was significant (I^2^ = 91.63%). Based on an Egger’s regression test for plot asymmetry, there was a significant publication bias (*p* = 0.001). A prevalence difference of 5.4% was detected, comparing autopsy (88.2%) and imaging (93.6%) studies (Table 1).

#### 3.4.5. C7 TF Entrance Prevalence

##### C7 TF Entrance of the AA-Originating LVAs

Four studies, including 181 AA-originating LVAs, were analyzed. The prevalence of the C7 TF entrance was 3.95% (CI: 1.1–6.8, *p* = 0.006), shown in Table 3. The heterogeneity was most likely not important (I^2^ = 0%). The publication bias was not significant (*p* = 0.173).

##### C7 TF Entrance of the SA-Originating LVAs

**Figure 21:**
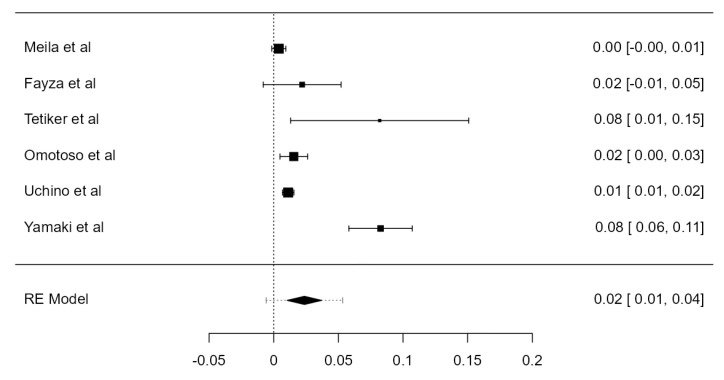
Forest plot—C7 TF entrance of the SA-originated LVAs.

3809 SA-originating LVAs from six articles were included. The prevalence of the C7 TF entrance was 2.37% (CI: 1.0–3.7, *p* < 0.001). The heterogeneity (I^2^ = 88.75%) and the publication bias (*p* < 0.001) were significant (Figure 21).

##### C7 TF Entrance of the LVAs

**Figure 22:**
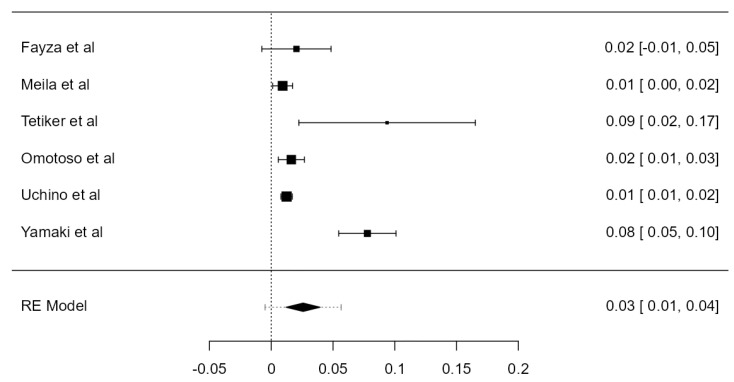
Forest plot—C7 TF entrance of the LVAs.

Six articles evaluating 4027 LVAs were evaluated. The prevalence of the C7 TF entrance was 2.58% (CI: 1.2–4.0, *p* < 0.001), shown in Figure 22. The heterogeneity was significant (I^2^ = 86.17%). The publication bias was significant (*p* = 0.002).

##### C7 TF Entrance of the RVAs

**Figure 23:**
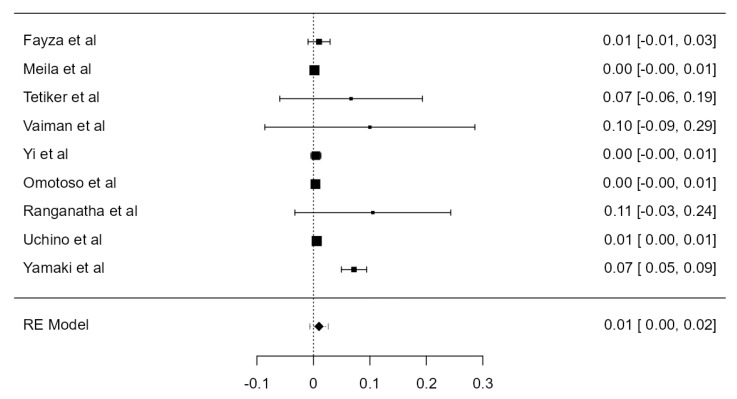
Forest plot—C7 TF entrance of the RVAs.

Nine papers, adding up to 4182 RVAs, were included to determine the C7 TF entrance prevalence of the right VAs. The overall value was 0.994% (CI: 0.3–1.7, *p* = 0.006), as in Figure 23. The heterogeneity was substantial (I^2^ = 81.03%). The publication bias, determined using Egger’s regression test for plot asymmetry, was significant (*p* < 0.001).

##### C7 TF Entrance

**Figure 24:**
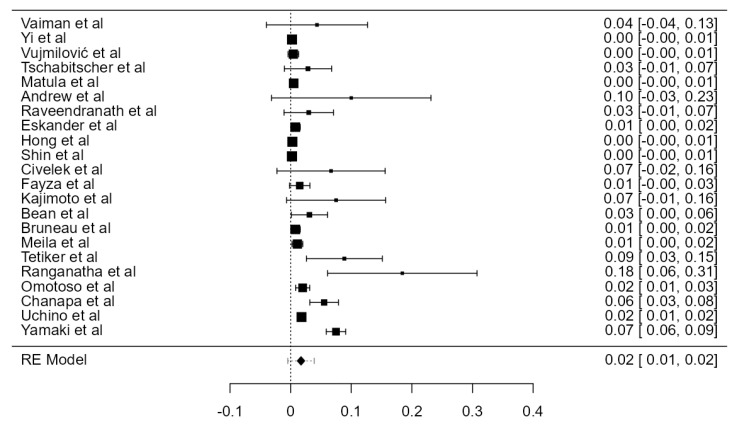
Forest plot—C7 TF entrance.

Twenty-two papers, adding up to 9159 VAs, were included in the present analysis. The crude prevalence of the C7 TF entrance was 1.72% (CI: 1.1–2.3, *p* < 0.001), shown in Figure 24. The heterogeneity was significant (I^2^ = 86.2%). The publication bias was significant (*p* < 0.001). A major prevalence difference was discovered between autopsy (5.42%) and imaging (0.8%) studies (Table 1).

### 3.5. Prevalence of the VA Course

#### 3.5.1. Tortuosity

##### Overall Tortuosity

**Figure 25:**
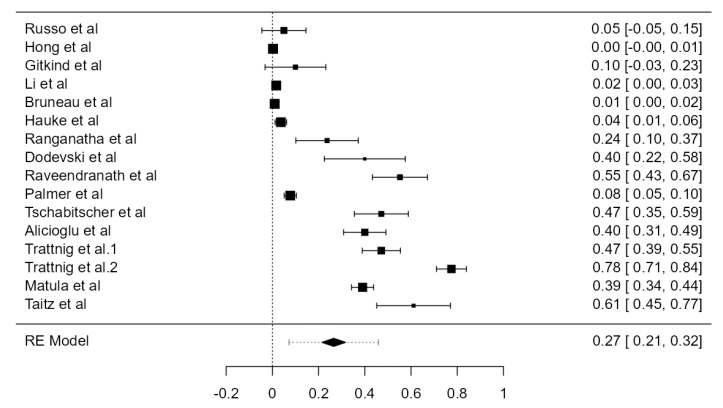
Forest plot—Tortuosity prevalence.

Fifteen original papers, summing 3149 VAs, were examined to determine the crude tortuosity prevalence of the V1 and V2 segments. Two articles reported different values for “significant” or “high” tortuosity [60,69]. These values were added up to the tortuosity value and analyzed altogether. The prevalence was estimated at 26.6% (CI: 21.4–31.7, *p* < 0.001), as represented in Figure 25. The heterogeneity was significant (I^2^ = 98.8%). The publication bias, based on Egger’s regression test for plot asymmetry, was significant (*p* < 0.001). A prevalence difference of 5.2% was found while comparing autopsy (31.0%) and imaging (25.8%) articles (Table 1).

##### Coronal Tortuosity Prevalence

Three papers, adding up to 380 tortuous VAs, were included in the analysis of coronal tortuosity prevalence. The determined value is 16.6% (CI: 8.4–24.8, *p* < 0.001), as in Table The heterogeneity was substantial (I^2^ = 78.87%). The publication bias was significant (*p* < 0.001).

##### Sagittal Tortuosity Prevalence

The sagittal tortuosity prevalence was 22.8% (CI: 17.6–28.0, *p* < 0.001). The heterogeneity was most likely not important (I^2^ = 27.87%). The publication bias, based on Egger’s regression test for plot asymmetry, was not significant (*p* = 0.058) (Table 3).

##### Transverse Tortuosity Prevalence

The transverse tortuosity prevalence was the highest, with a percentage of 30.1 (CI: 18.2–42.0, *p* < 0.001), shown in Table 3. The heterogeneity was substantial (I^2^ = 84.63%). The publication bias was significant (*p* = 0.008).

#### 3.5.2. Straight V1 Segment Prevalence

Three articles, including 430 VAs, were analyzed. The prevalence of a straight pretransverse segment was 45.2% (CI: 35.7–54.6, *p* < 0.001). The heterogeneity was substantial (I^2^ = 74.14%). The publication bias was not significant (*p* = 0.605) (Table 3).

### 3.6. VA Dominance

**Figure 26:**
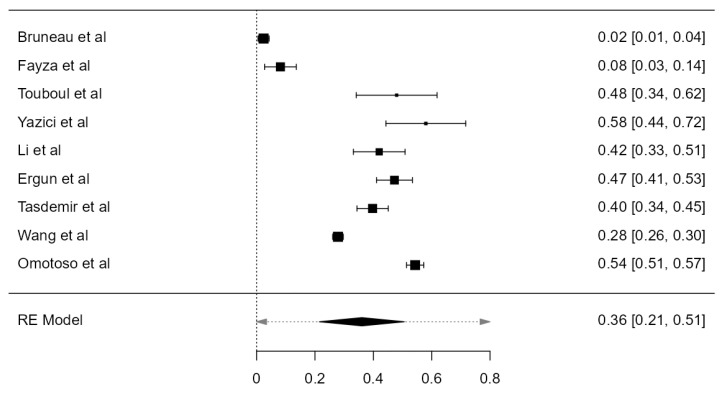
Forest plot—LVA dominance prevalence.

**Figure 27:**
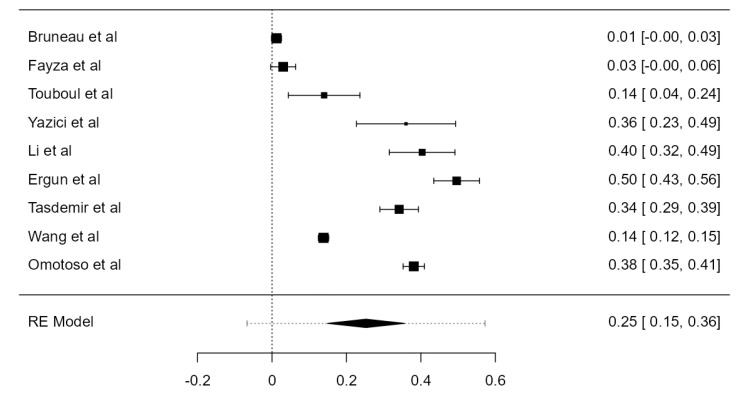
Forest plot—RVA dominance prevalence.

Nine studies, summing 4623 VAs, were included in the current analysis to determine the LVA/RVA dominance crude prevalence. Considering the LVA, the prevalence was 36.1% (CI: 21.5–50.6, *p* < 0.001), higher in comparison with RVA prevalence, which was 25.3% (CI: 14.9–35.6, *p* < 0.001), shown in Figure 26 and Figure 27. The heterogeneity was significant (I^2^ = 99.27%, 99.08%, respectively). The publication bias was not significant (*p* = 0.150, 0.095, respectively).

### 3.7. VA Hypoplasia Prevalence

**Figure 28:**
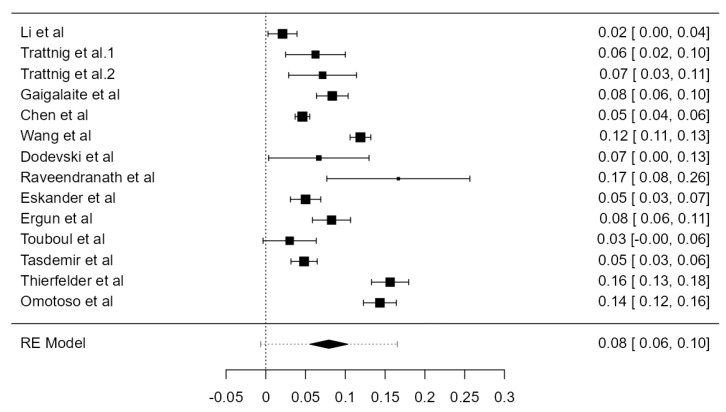
Forest plot—Hypoplasia prevalence.

Fourteen articles were included, adding up to 9570 VAs. The crude prevalence of such a condition was 7.94% (CI: 5.6–10.3, *p* < 0.001), shown in Figure 28. The heterogeneity was significant (I^2^ = 94.42%). The publication bias was not significant (*p* = 0.425). No major difference was detected between autopsy and imaging articles (Table 1).

## 4. Discussion

### 4.1. Anatomical Incidence

Considering the VA topography and morphology, the results are in accordance with the information stated in different anatomical textbooks [76,77]. The most prevalent site of origin for both LVA and RVA is the SA with a frequency of 94.1%, respectively 99.9%. Regarding the entrance in a certain TF, most VAs (92%) enter at the level of the sixth cervical vertebra, followed by C5, C7, C4, and, less frequently, C3. AA-originated LVAs present the highest frequency of entrance into the fifth cervical vertebra TF (69.2%), then C4, C6, C3, and C7. This result contradicts Morris [78] who stated that AA-originated LVAs commonly enter the TF of the fourth cervical vertebra. Roughly one out of four VAs presented a form of tortuosity, the transverse tortuosity having the highest prevalence. From the current meta-analysis, it also resulted in a higher frequency of LVA dominance compared to RVA, which suggests that a greater volume of blood is conducted through the LVA to the brain. Of significant clinical importance is the fact that LVA damage may lead to deterioration of the brain’s blood supply [79,80]. As regards hypoplastic VAs, the prevalence is relatively low; roughly 8% of the vessels were reported with a diameter lower than 3.5 mm. VA origin has been widely evaluated and it is acknowledged that the most frequent origin on each side is from the SA. Arteries originating from any other site are considered aberrant [81,82]. LVAs most frequent anatomic variant is the direct aortic origin, with a prevalence of 2.4–5.8% [83]. In the current study, we determined a prevalence of 4.81% of the AA-originated LVA, which conforms with the range 2.4–4.8% [83] reported by Adachi et al. [19], Yamaki et al. [73], Uchino et al. [69], etc. Other origins have been reported: left external carotid artery, thyrocervical trunk, carotid bulb, left common carotid artery, but the frequency is extremely low (0.1–0.2%) [82] and there is an insufficient amount of prevalence studies that reported these variants. Concerning RVA’s abnormal origin, the brachiocephalic trunk was the most frequent with a prevalence of 0.539%, followed by RCCA and AA origin. As these variants are remarkably uncommon, they have been mostly communicated as case reports [84,85,86,87,88,89].

As documented in *Bergman’s Comprehensive Encyclopedia of Human Anatomic Variation*, VAs could originate from any carotid artery, common, internal, or external [90]. However, these anatomical possibilities correspond to persistent proatlantal arteries which are rarely found [91] and could appear as either V3 segments with carotid origin, with normoplastic or hypoplastic V1–V2 VAs supplied from the SAs [92], or as proatlantal arteries with absent VAs [93].

**Figure 29:**
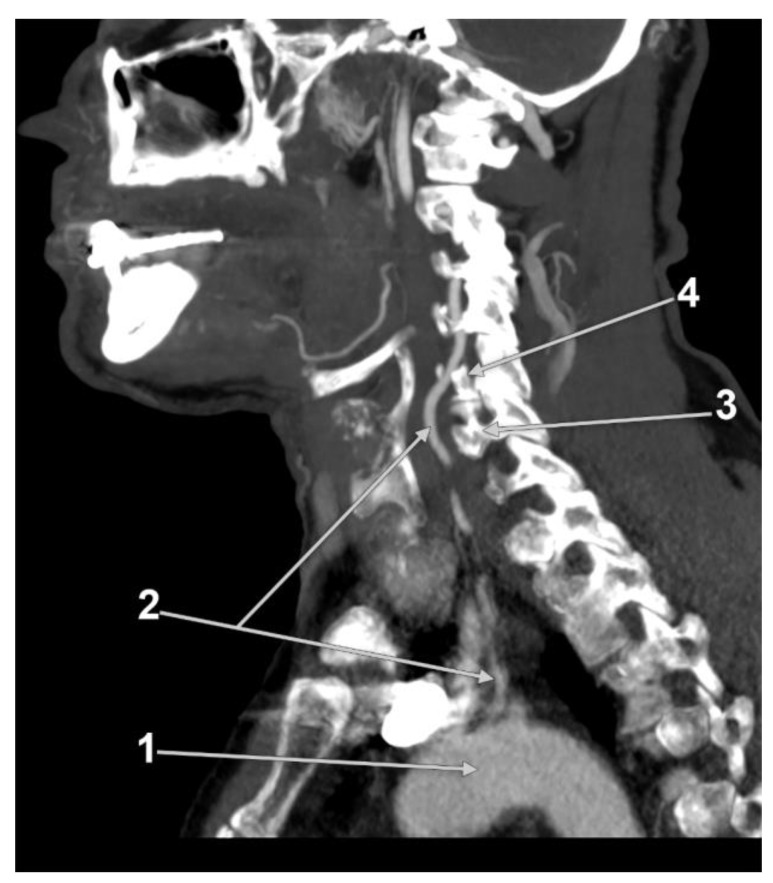
Computed tomography angiogram. Sagittal slice through the left vertebral artery originating the aortic arch and entering the transverse foramen of the fifth cervical vertebra. Original evidence: 1. aortic arch; 2. left vertebral artery; 3. transverse process of sixth cervical vertebra; 4. transverse process of fifth cervical vertebra.

The V2 segment of the VA is commonly situated between C6 and C2. An anomalous entrance is frequently correlated with an abnormal origin [69]; the most prevalent variant is the AA-originated LVA entering the fifth cervical TF in 78.6% (Figure 29) [72], compared to 69.2% stated in the current study. Li et al. [50] confirmed this variant, as the LVA with an anomalous origin entered the TF at C5 and presented a sinuous course. Tardieu et al. [94] also found that AA-originating LVAs were more likely to enter the TF more cranially than C6 and to adopt a more medial course over the cervical vertebral bodies.

**Figure 30:**
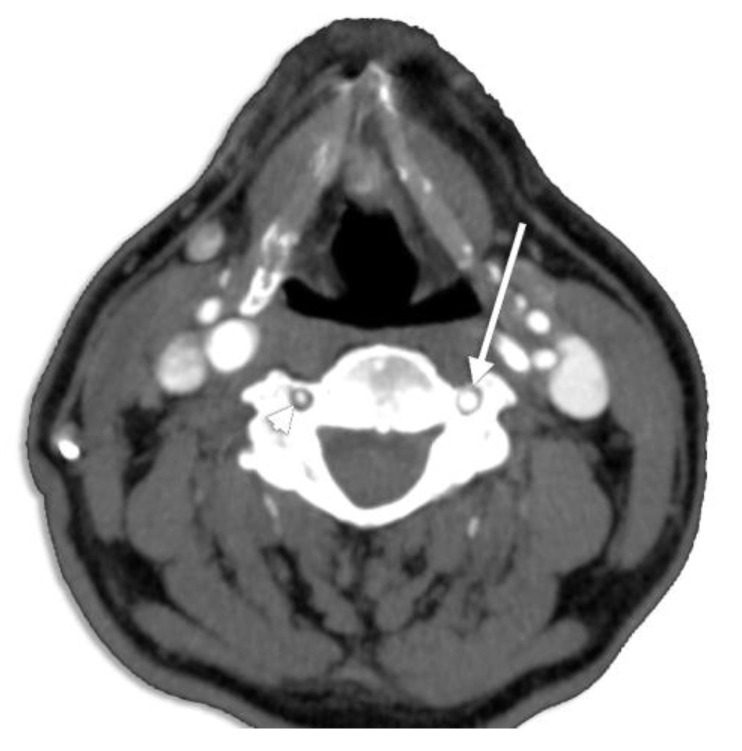
Axial CT slice at the level of C4 viewed inferiorly. Right vertebral artery hypoplasia (arrowhead). Normoplastic left vertebral artery (arrow).

Gantwerker et al. [95] correlated VA’s diameter with the size of the TF; thus, a small TF may presume a hypoplastic or even an aplastic vessel (Figure 30). Hypoplastic VAs have been widely analyzed for their clinical appliance, being a predisposing factor for posterior circulation ischemia [96]. George and Laurian [97] reported a frequency of 5.7% for LVA hypoplasia and 8.8% for hypoplastic RVAs, without mentioning the hypoplastic segment (V1–V4) and including also the atretic arteries.

**Figure 31:**
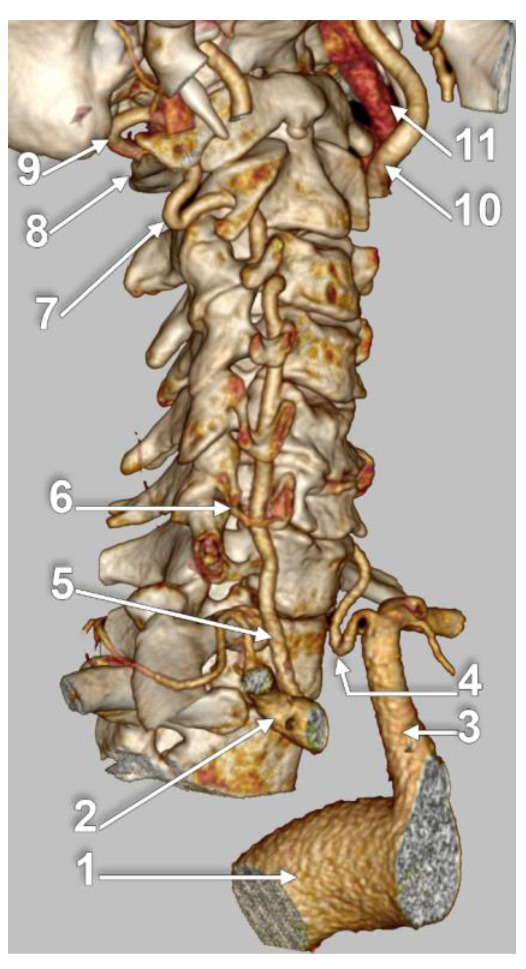
Angio-CT, three-dimensional volume rendering of the vertebral arteries (VAs). Right infero-lateral view. Kinked V1 segment of left VA and V2 segment of right VA: 1. aortic arch; 2. right subclavian artery; 3. left subclavian artery; 4. inferiorly kinked V1 segment of left VA; 5. right VA; 6. right transverse process of C6 vertebra; 7. laterally kinked V2 segment of right VA, in the C1–C2 intertransverse space; 8. posterior arch of atlas; 9. V3 segment of right VA; 10. left internal carotid artery; 11. left internal jugular vein.

VA loops occur most frequently in the V2 segment (90.5%), followed by V1 (7.6%) and V3 (1.9%) (Figure 31), with the highest occurrence in females, aged between fifty and seventy years old [98].

It worth discussing here that attributing other meanings to generally accepted labels, such as V1, V2, or V3, would lead to misunderstandings and erroneous assumption of the reported data. Kiresi et al. [99] and Cacciola et al. [100] described the anatomical area between C3 and C2 TF as V1, C2 TF to C1 TF as V2, and C1 to dural entry point as V3. Fisher et al. [101] describe the V2 segment as the VA part running between C2–C3 and dura mater. This assignment was also used by Lang et al. [102]. Considering the convention of term usage, V1–V4 labels describe the four segments of the VA, from the SA to the origin of the basilar artery [7,8,103,104,105], as detailed in the Introduction. We strongly advise authors to avoid term reassignments.

### 4.2. Embryology

During embryogenesis, from the dorsal aspect of the aorta arise seven intersegmental branches, referred to as C1–CUsually, the first part of the VA develops from the dorsal branch of the seventh cervical intersegmental artery. Longitudinal communications of the postcostal anastomoses form the second part of the VA [106,107].

The AA-originating LVA is considered to result from a persisting sixth cervical intersegmental artery, which fails to disappear. The blood flows directly from the AA to the C6 intersegmental artery, creating the AA origin of the LVA [108,109]. Increased resorption of embryonic tissue of the LSA between the AA and LVA origin may also lead to an anomalous origin [110].

It should be pointed out the significance of two persistent arteries, proatlantal and hypoglossal artery, which along with the trigeminal and otic arteries represent the deficient involution of embryonic vascular channels [111]. Hypoplasia of the VA, uni- or bilateral, is correlated with a prevalence of around 50% with the persistence of the proatlantal artery. It usually corresponds to the V3 segment of the VA, originates from the internal or the external carotid artery and enters the skull through the foramen magnum [111,112,113]. Persistence of the hypoglossal artery is related to VA aplasia, with a prevalence of 0.02–0.1%. A persistent hypoglossal artery originates from the internal carotid artery and enters the skull via the hypoglossal canal [113,114]. The entrance in the skull via different foramina also represents a useful criterion in distinguishing the two arteries [115].

### 4.3. Clinical Relevance

Patients with variation in VA origin are exposed to an increased risk in AA or esophageal surgery, if the case is not previously documented. “Vertebral arteria lusoria” represents a VA originating from the AA, distal to the origin of the left SA, commonly with a retroesophageal course. This anatomical variant has been seldom reported in the literature [83,84,116,117]. Verin et al. reported the case of a lusoria VA entering the fourth cervical vertebra [118], and Meila et al. [52] reported the TF entrance of such a vessel at CThis condition was associated with life-threatening risk during esophageal surgery, as accidental injury to this vessel may lead to hemomediastinum or even neurological impairment [119].

Variation in the entrance into the TF should also be presurgical detailed, as it may expose the patient to risks during anterior neck surgery [51] or dissection during neck rotation [120].

Ignoring the VA anatomical variability could lead to its injury and life-threatening hemorrhage during anterior neck surgery, for instance, thyroidectomy or excision of a pharyngeal diverticulum, as the VA could be damaged while attempting ligation of the inferior thyroid artery [121].

Gluncic et al. [87] stated that atherosclerosis frequently affects the extracranial part of the VA. The origin of the VA is therefore predisposed to subsequent stenosis. Bernard and Dettori [122] presumed that an abnormal VA origin, distribution, or caliber favors cerebral disorders by altering cerebral hemodynamics. The study was conducted on two cases, so their hypothesis may not be generally applicable. Contrarily, Einstein et al. [33] stated that an aberrant LVA may have no vascular consequences, unless being compromised during AA surgery.

Tortuosity and hypoplasia of the VA have a considerable impact on posterior circulation infarctions and atherosclerosis [123,124]. The cause leading to tortuous vessels was speculated by Morris et al. [125] as being a thinner, fragile wall that displays as a meandering vessel. Another etiology proposed by Choi et al. [126] is that the VA adapts as the cervical spine develops a tortuous route due to osteoarthritis. Congenital anomalies of the craniovertebral junction [127] and trauma [128] were also suggested to determine tortuosity. Tortuosity is also linked with vascular vertigo [129] but only if one segment of the VA is affected; if no vascular risk factors exist, symptoms may not appear [130]. A twisted vessel is at higher risk of iatrogenic injuries, as it does not pursue a common course [131]. Thus, a pre-surgical imagistic recognition of the anatomical peculiarities of the VA is fundamental.

### 4.4. Study Limitations

Although, to our knowledge, this could be regarded as the most extensive meta-analysis study on the VA topic that comprehensively includes numerous anatomical aspects, we should admit that it has several limitations. First, as with any other meta-analysis, the present paper includes a great variety of studies, regardless of the patient’s pathologies, geographic area, number of patients, study types, etc. To minimize this major heterogeneity, we added exclusion criteria, as the removal of papers with patients suffering from vascular disease or studies using less than 10 subjects. Second, considering the Origin and TF Entrance sections, the analyses included all the available studies on the specific anatomic variant. As the total number of evaluated arteries varied, the results are not comparable to each other. Considering the definition of a systematic review or a meta-analysis, we processed the available data in the literature; thus, the assessment of both crude and standardized prevalence was not attainable for each variant analysis. Our results are, thus, highly influenced by the reported results. To diminish any risk of affecting the outcomes of the present study due to biased results published by other authors, we filtered each article rigorously, applying the third exclusion criteria.

### 4.5. Small-Study Effect

The term “small-study effect” was proposed by Sterne et al. to express the observation that smaller studies typically produce greater effect sizes [132]. According to Yurasakpong et al., the small-study effect, which denotes publication bias, is a frequent issue, particularly in anatomical systematic reviews and meta-analyses [133]. Analyses with high standard errors (SE) and low sample size are more likely to report higher prevalence. Funnel plots of effect size against sample size were used to detect publication bias. The asymmetrical plots may suggest the potential impact of the small-study effect and publication bias on the results of the interested determinations.

## 5. Conclusions

The anatomy of the VA is extensively variable. The SA origin of the VA has a prevalence of 94.1%. The entrance of the VA at the sixth transverse foramen prevails in 92.0% of cases. Tortuosity (26.6%) and hypoplasia (7.94%) were also documented. Tortuosity in the transverse plane was the most frequent (30.1%), related to sagittal (22.8%) and coronal (16.6%) tortuosity. A straight V1 segment of the VA was prevalent in 45.2% of cases. Considering the VA dominance, LVA was dominant in 36.1% of cases, compared to RVA dominance, found in 25.3% of cases.

## Figures and Tables

**Table 1:** Determined prevalence for anatomical characteristics based on study type (autopsy or imaging).

Anatomical Characteristics	Study Type	Total No. of Articles	Prevalence (%)	Statistical Significance (*p* < 0.05)	Heterogeneity (%)	Significant Publication Bias (*p* < 0.05)
LVA_AA	Autopsy	16	5.47	Yes	31.05	Yes
LVA_AA	Imaging	19	4.59	Yes	73.16	No
LVA_SA	Autopsy	15	94.5	Yes	33.29	Yes
LVA_SA	Imaging	16	93.5	Yes	97.31	Yes
RVA_SA	Autopsy	9	99.8	Yes	19.18	Yes
RVA_SA	Imaging	14	99.9	Yes	0	Yes
C4_TF entrance	Autopsy	8	0.7	Yes	0	No
C4_TF entrance	Imaging	17	1.45	Yes	61.08	Yes
C5_TF entrance	Autopsy	13	4.37	Yes	72.12	No
C5_TF entrance	Imaging	17	8.36	Yes	99.34	Yes
C6_TF entrance	Autopsy	17	88.2	Yes	92.46	No
C6_TF entrance	Imaging	19	93.6	Yes	91.53	No
C7_TF entrance	Autopsy	9	5.42	Yes	44.21	No
C7_TF entrance	Imaging	13	0.8	Yes	71.82	Yes
Tortuosity	Autopsy	8	31.0	Yes	96.84	No
Tortuosity	Imaging	8	25.8	Yes	99.92	No
Hypoplasia	Autopsy	3	7.63	No	89.36	Yes
Hypoplasia	Imaging	11	8.16	Yes	94.72	No

**Table 2:** Studies included in the analysis.

Reference & Year	Study Type	Number of Specimens	Area of Interest	NOS-Modified Score
Adachi 1928 [19]	Autopsy	516	Origin/TF entrance	5
Alicioglu 2015 [20]	Imaging	110	Origin/Tortuosity	5
Yazici 2018 [21]	Imaging	100	Dominance	4
Gitkind 2014 [22]	Autopsy	20	TF entrance/Tortuosity	5
Bean 1905 [23]	Autopsy	129	Origin/TF entrance	6
Bergman 2012 [24]	Autopsy	693	Origin	5
Berko 2009 [25]	Imaging	1000	Origin	7
Bhatia 2005 [26]	Autopsy	81	Origin	4
Bruneau 2006 [8]	Imaging	500	TF entrance/Dominance/Tortuosity	5
Budhiraja 2013 [27]	Autopsy	52	Origin	5
Taitz 1991 [28]	Autopsy	36	Tortuosity	5
Cavdar 1996 [29]	Autopsy	24	TF entrance	5
Chanapa 2012 [30]	Autopsy	362	TF entrance	4
Lin 2018 [31]	Imaging	2437	Origin/TF entrance	6
Dodevski 2011 [32]	Imaging	60	Origin/TF entrance/Tortuosity/Hypoplasia	5
Einstein 2016 [33]	Autopsy	27	Origin	4
Civelek 2007 [34]	Autopsy	30	TF entrance	4
Ergun 2016 [35]	Imaging	508	Origin/Hypoplasia/Dominance	6
Eskander 2010 [36]	Imaging	500	TF entrance/Hypoplasia	5
Fayza 2019 [37]	Imaging	200	Origin/TF entrance/Dominance	5
Gaigalaite 2016 [38]	Imaging	742	Hypoplasia	6
Güvençer 2006 [39]	Imaging	24	TF entrance	4
Tetiker 2014 [40]	Imaging	79	Origin/TF entrance	4
Hauke 1973 [41]	Imaging	218	Tortuosity	5
Heary 1996 [42]	Imaging	16	TF entrance	4
Hong 2008 [43]	Imaging	700	TF entrance/Tortuosity	6
Ikegami 2007 [44]	Autopsy	50	Origin/TF entrance	4
Jakanani 2010 [45]	Imaging	861	Origin	7
Johnson 1995 [46]	Autopsy	20	TF entrance	5
Kajimoto 2007 [47]	Autopsy	40	TF entrance	5
Kim 2017 [48]	Imaging	476	Origin/TF entrance	5
Komiyama 2001 [49]	Imaging	1567	Origin	6
Li 2019 [50]	Autopsy	238	Origin/Tortuosity/Hypoplasia	6
Matula 1997 [51]	Imaging	402	TF entrance/Tortuosity	6
Meila 2012 [52]	Imaging	1083	Origin/TF entrance	6
Muller 2011 [53]	Imaging	2033	Origin	7
O’Malley 2018 [54]	Autopsy	24	Origin	5
Omotoso 2021 [55]	Imaging	1108	Origin/TF entrance/Hypoplasia/Dominance	5
Palmer 1980 [56]	Imaging	400	Tortuosity	5
Patil 2012 [57]	Autopsy	75	Origin	5
Ranganatha 2006 [58]	Autopsy	38	TF entrance/Tortuosity	4
Raveendranath 2014 [59]	Autopsy	66	Origin/TF entrance/Tortuosity/Hypoplasia	5
Russo 2011 [60]	Autopsy	20	TF entrance/Tortuosity	5
Vujmilović 2018 [61]	Imaging	224	Origin/TF entrance	5
Schmitt 2019 [62]	Autopsy	10	TF entrance	5
Shin 2014 [63]	Imaging	920	TF entrance	6
Shin 2008 [64]	Autopsy	28	Origin	6
Tasdemir 2022 [1]	Imaging	644	Origin/TF entrance/Hypoplasia/Dominance	6
Thierfelder 2014 [65]	Imaging	934	Hypoplasia	6
Touboul 1986 [66]	Imaging	100	TF entrance/Hypoplasia/Dominance	6
Toyoharu 1991 [67]	Autopsy	144	Origin	5
Trattnig 1993 [17]	Autopsy	140	Tortuosity/Hypoplasia	5
Trattnig 1993 [17]	Imaging	160	Origin/Tortuosity/Hypoplasia	6
Tschabitscher 1991 [15]	Autopsy	70	TF entrance/Tortuosity	5
Tubbs 2009 [68]	Autopsy	20	TF entrance	4
Uchino 2013 [69]	Imaging	4574	Origin/TF entrance	6
Vaiman 2010 [18]	Imaging	23	Origin/TF entrance	5
Vorster 1998 [70]	Autopsy	120	Origin	5
Wang 2016 [71]	Imaging	2370	Origin/Hypoplasia/Dominance	4
Woraputtaporn 2019 [72]	Autopsy	532	Origin/TF entrance	6
Yamaki 2006 [73]	Autopsy	1029	Origin/TF entrance	6
Chen 2009 [74]	Imaging	2000	Hypoplasia	6
Yi 2022 [75]	Imaging	446	Origin/TF entrance	6

**Table 3:** Determined prevalence for anatomical characteristics reported in fewer than 5 articles.

Anatomical Characteristic	Total No. of Articles	Total No. of Cases	Prevalence (%)	Statistical Significance (*p* < 0.05)	Heterogeneity (%)
RVA_AA	4	2992	0.1	No	11.7
RVA_RCCA	4	4368	0.126	Yes	0
RVA_BCT	3	848	0.539	No	73
C3_TF ENTRANCE_LVA_AA	3	40	8.11	No	0
C4_TF ENTRANCE_LVA_SA	4	2736	0.337	No	39.11
C7_TF ENTRANCE_LVA_AA	4	181	3.95	Yes	0
Coronal Tortuosity	3	380	16.6	Yes	78.87
Sagittal Tortuosity	3	380	22.8	Yes	27.87
Transverse Tortuosity	3	380	30.1	Yes	84.63
Straight V1	3	430	45.2	Yes	74.14

## Data Availability

Not applicable.

## Supplementary Materials

The following supporting information can be downloaded at: https://www.mdpi.com/article/10.3390/diagnostics13122036/s1, Figure S1: C3_TF_funnel sample size, Figure S2: C3_TF_funnel SE, Figure S3: C4_TF_LEFT_funnel sample size, Figure S4: C4_TF_LEFT_funnel SE, Figure S5: C4_TF_RIGHT_funnel sample size, Figure S6: C4_TF_RIGHT_funnel SE, Figure S7: C4_TF_funnel sample size, Figure S8: C4_TF_funnel SE, Figure S9: C5_TF_LEFT_SA_funnel sample size, Figure S10: C5_TF_LEFT_SA_funnel SE, Figure S11: C5_TF_LEFT_funnel sample size, Figure S12: C5_TF_LEFT_funnel SE, Figure S13: C5_TF_RIGHT_funnel sample size, Figure S14: C5_TF_RIGHT_funnel SE, Figure S15: C5_TF_funnel sample size, Figure S16: C5_TF_funnel SE.

## Abbreviations

AA	aortic arch
BCT	brachiocephalic trunk
C3_TF Entrance_LVA_AA	AA-originated LVA entering the TF at C3
C4_TF Entrance_LVA_SA	SA-originated LVA entering the TF at C4
C7_TF Entrance_LVA_AA	AA-originated LVA entering the TF at C7
CI	confidence interval
LSA/RSA	left/right SA
LVA/RVA	left/right VA
LVA_AA	AA-originated LVA
LVA_SA	SA-originated LVA
RCCA	right common carotid artery
RVA_AA	AA-originated RVA
RVA_BCT	BCT-originated RVA
RVA_RCCA	RCCA-originated RVA
RVA_SA	SA_originated RVA
SA	subclavian artery
TF	transverse foramen
VA	vertebral artery
