# Supplementary material for: The Vertebral Artery: A Systematic Review and a Meta-Analysis of the Current Literature
Source: Diagnostics (Basel). 2023 Jun 12;13(12):2036. doi: 10.3390/diagnostics13122036 (PMC10296927; doi:10.3390/diagnostics13122036)
Supplement: Supplementary file 1 [file diagnostics-13-02036-s001.zip › diagnostics-2413306-supplementary.pdf]

diagnostics

Supplementary Material

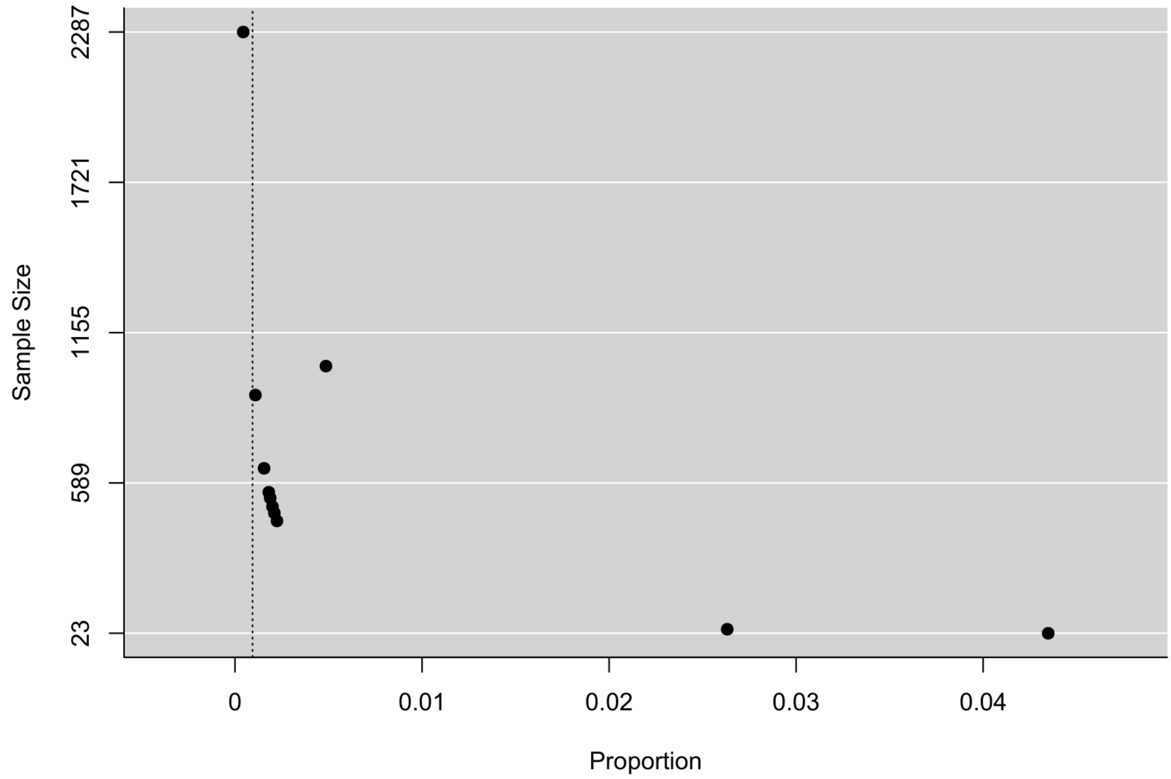

Figure S1. C3_TF_funnel sample size.

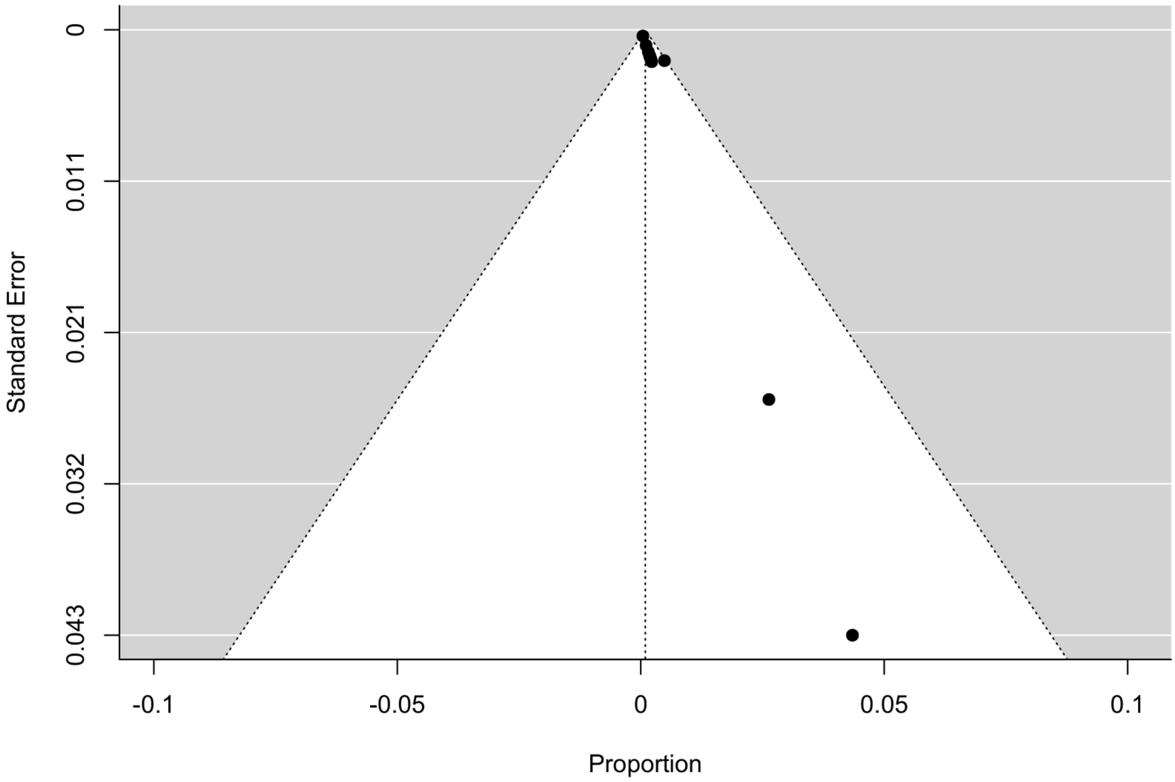

Figure S2. C3_TF_funnel SE.

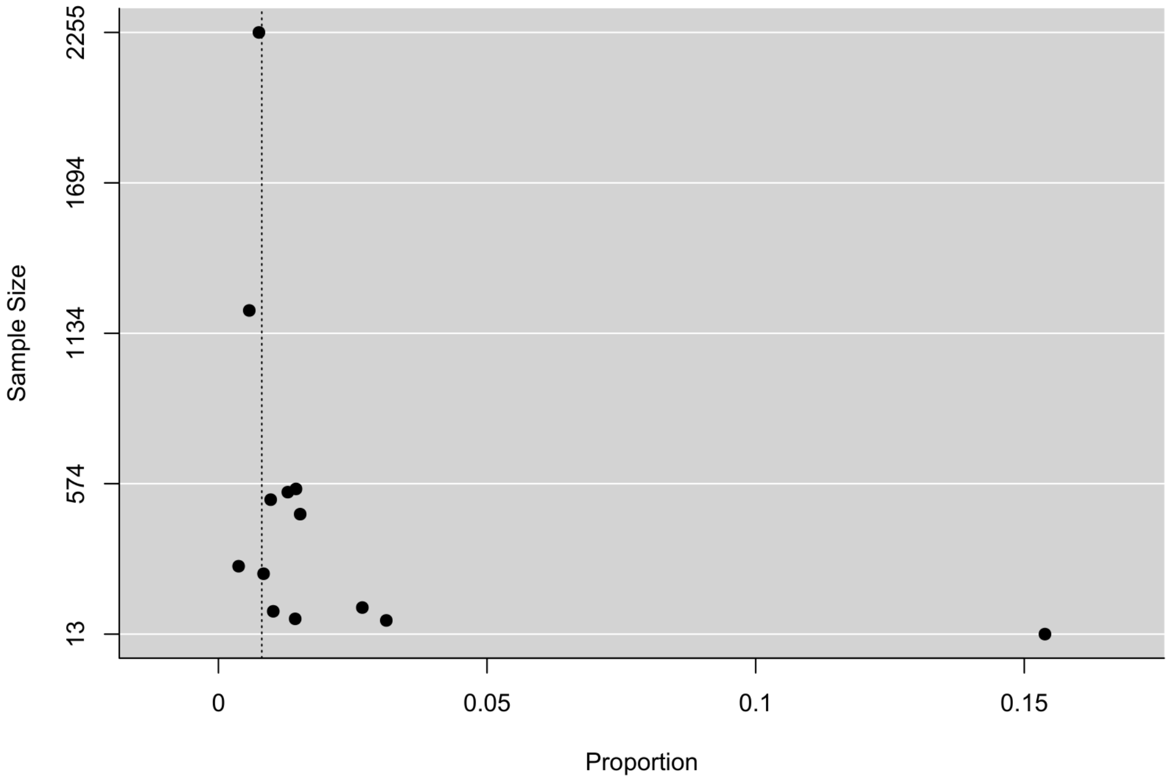

Figure S3. C4_TF_LEFT_funnel sample size.

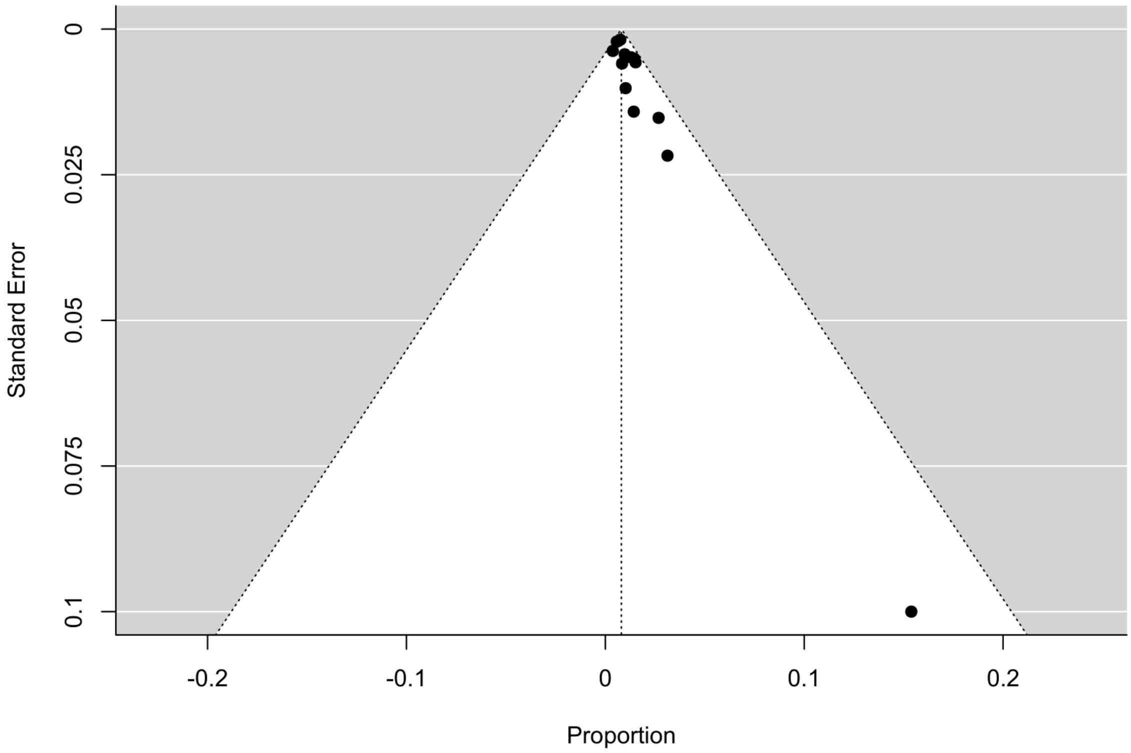

Figure S4. C4_TF_LEFT_funnel SE.

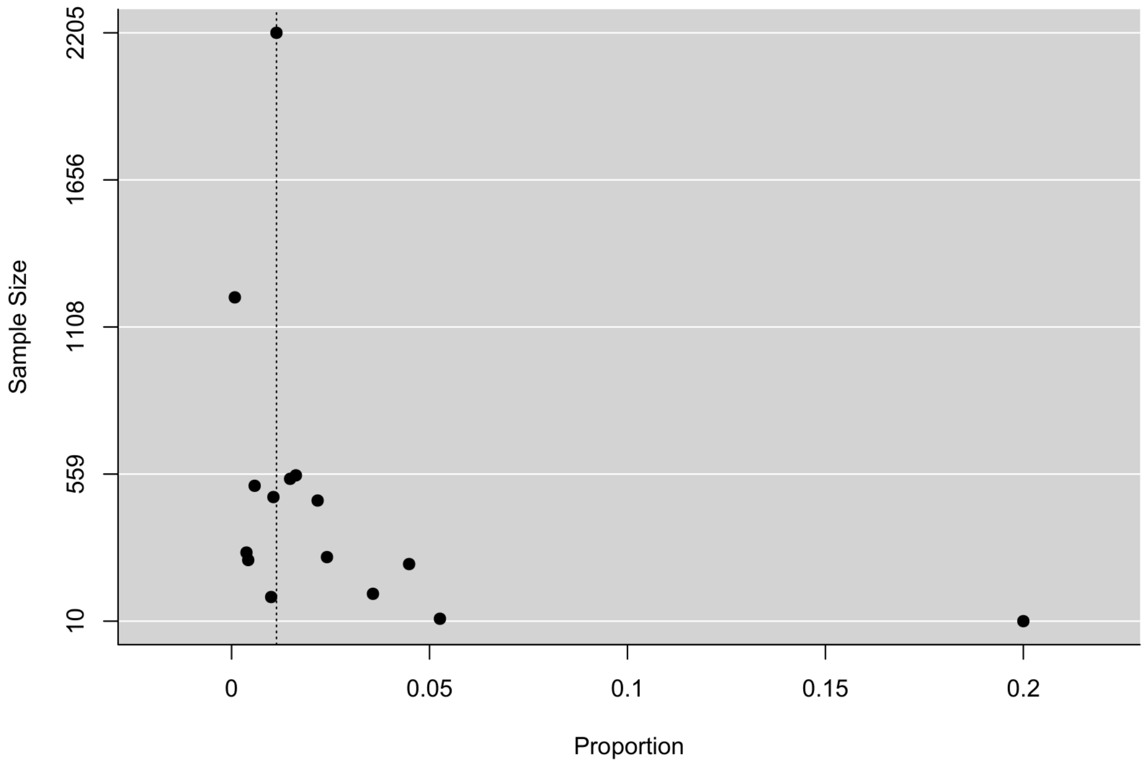

Figure S5. C4_TF_RIGHT_funnel sample size.

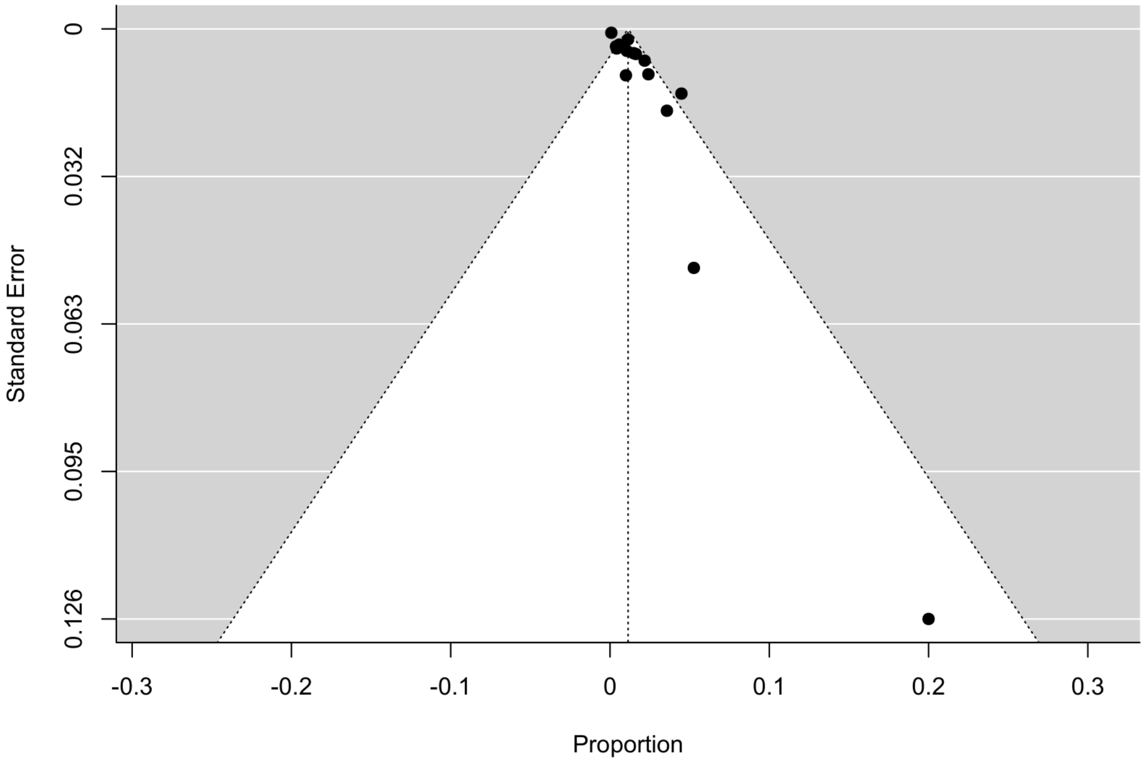

Figure S6. C4_TF_RIGHT_funnel SE.

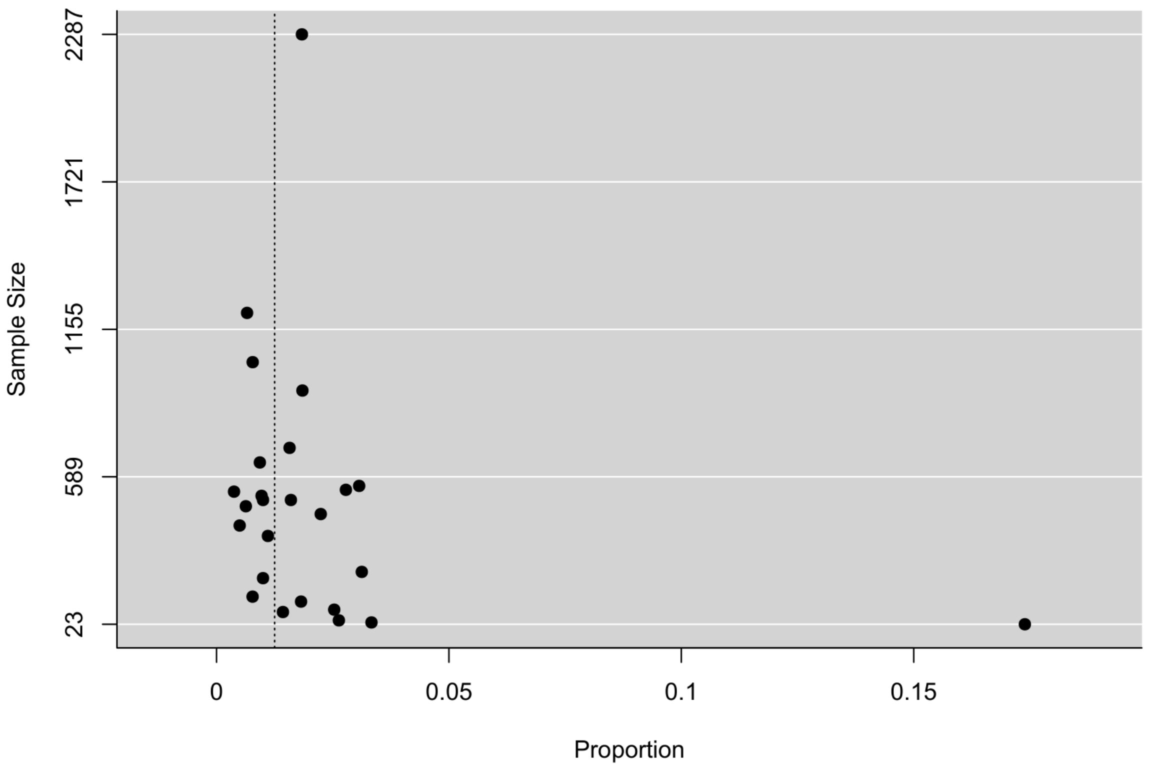

Figure S7. C4_TF_funnel sample size.

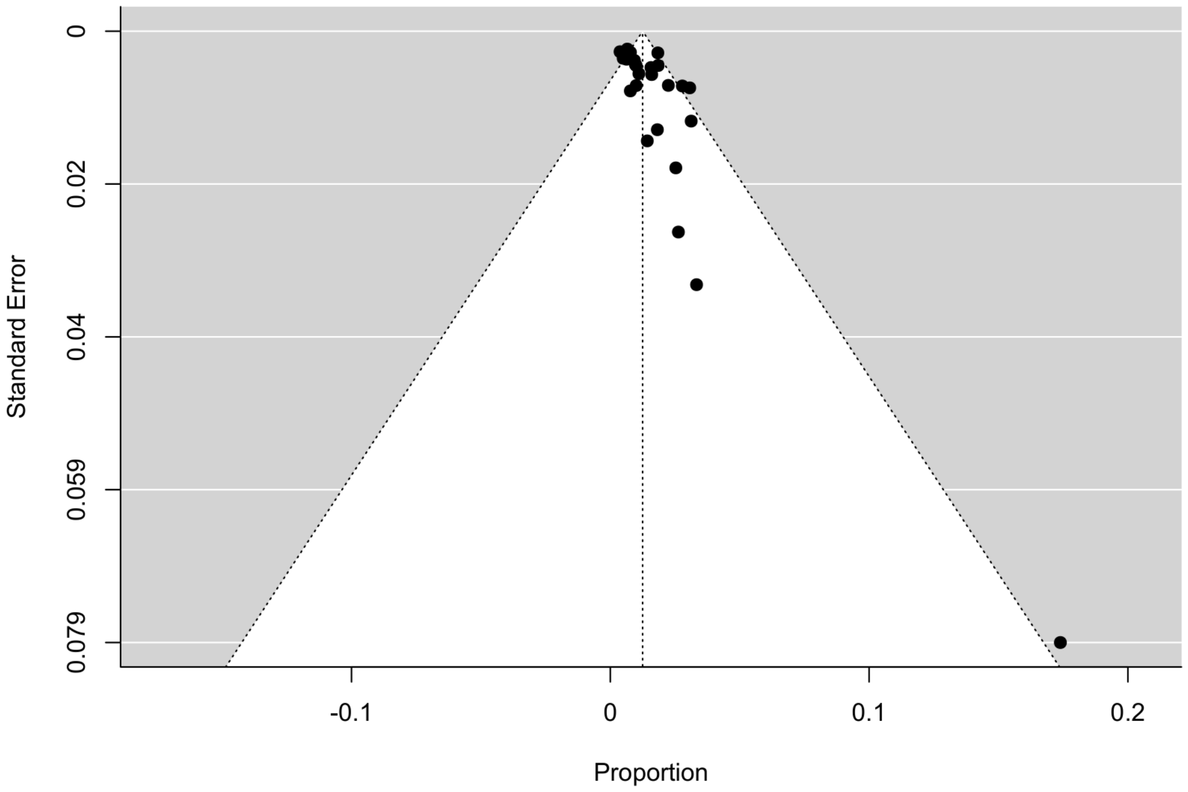

Figure S8. C4_TF_funnel SE.

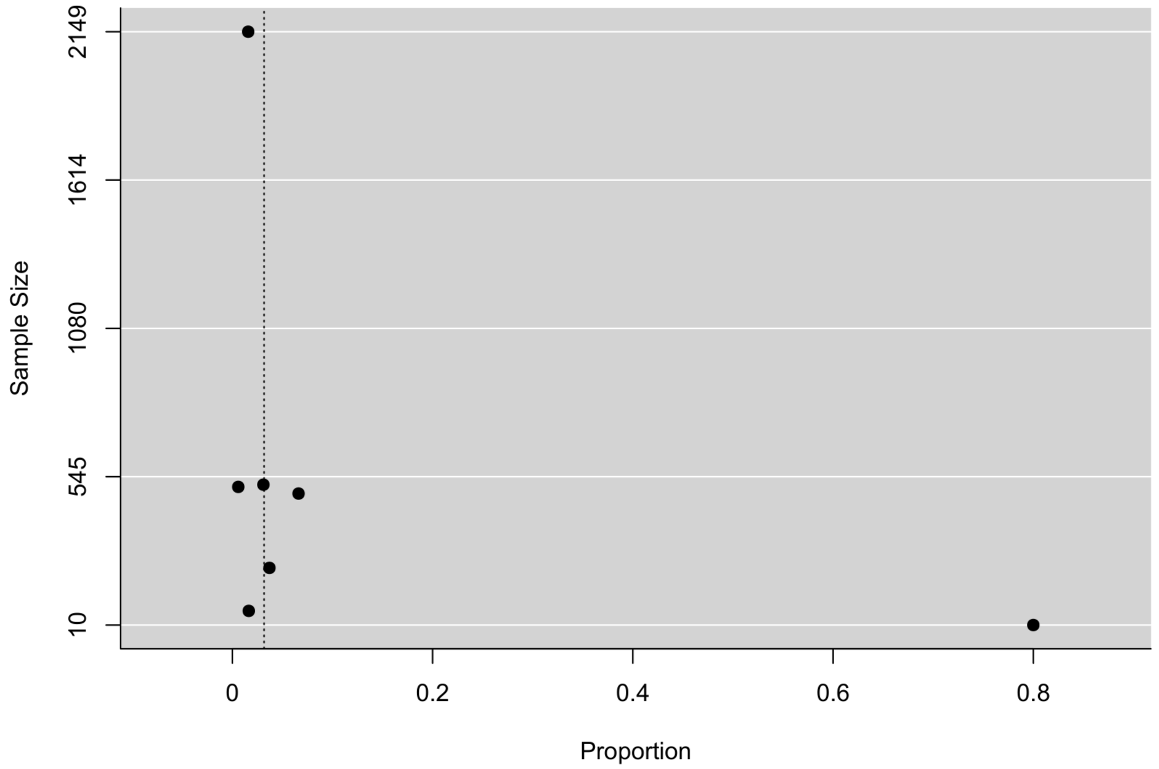

Figure S9. C5_TF_LEFT_SA_funnel sample size.

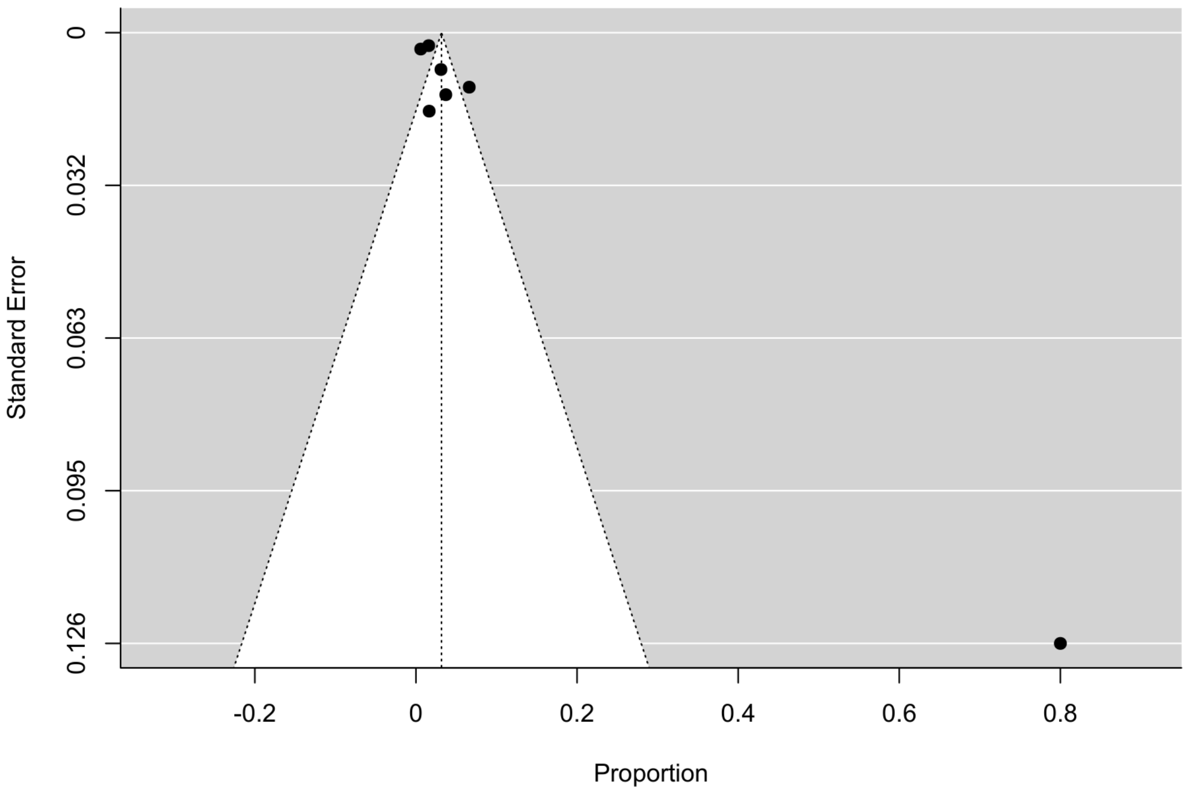

Figure S10. C5_TF_LEFT_SA_funnel SE.

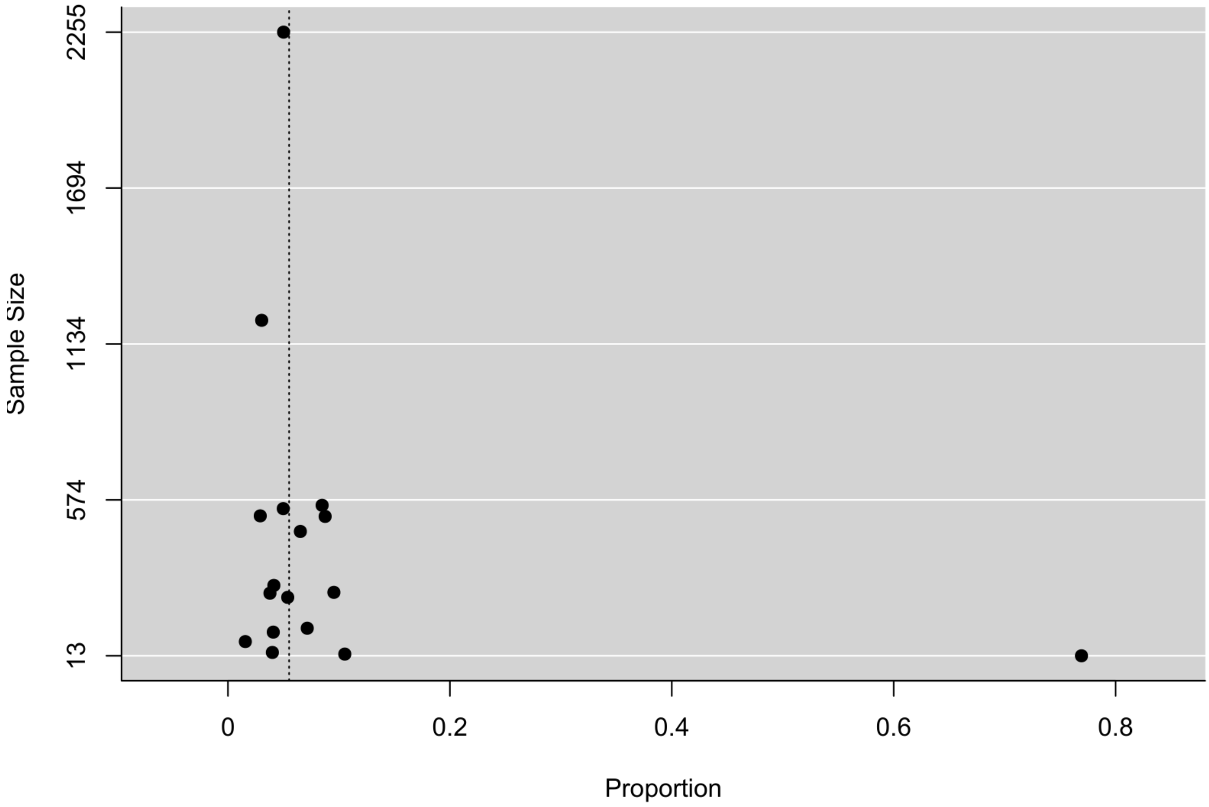

Figure S11. C5_TF_LEFT_funnel sample size.

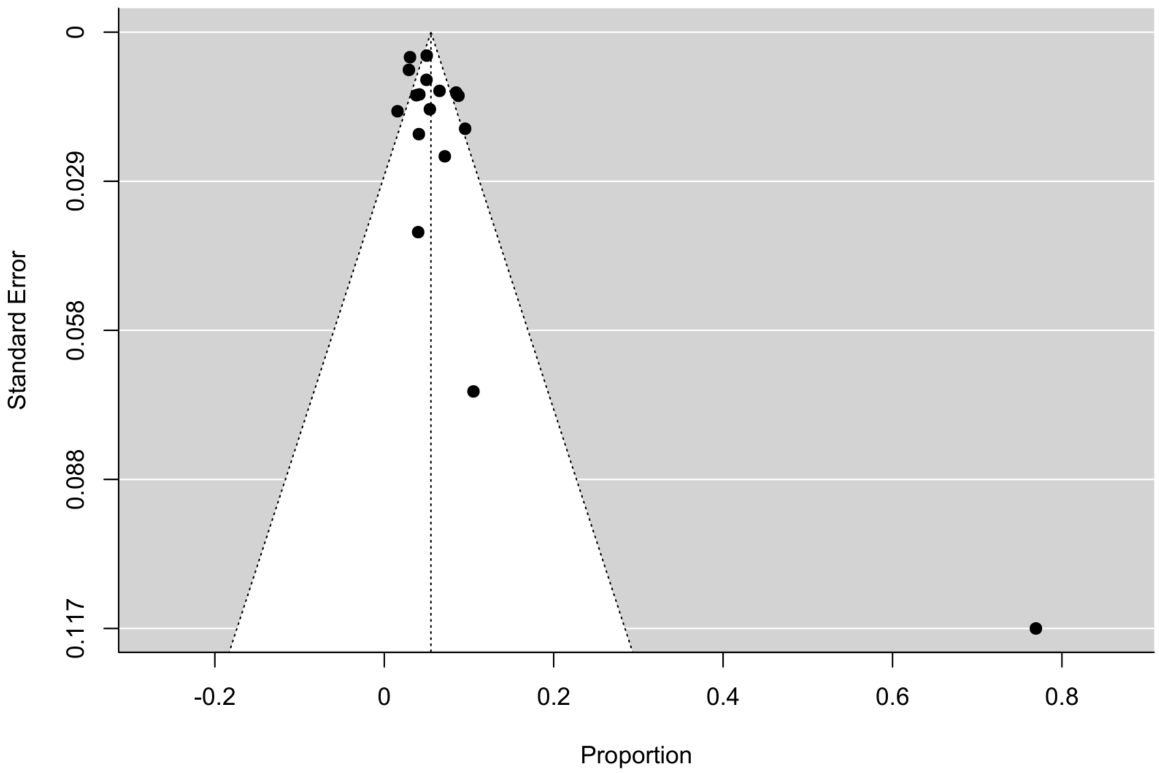

Figure S12. C5_TF_LEFT_funnel SE.

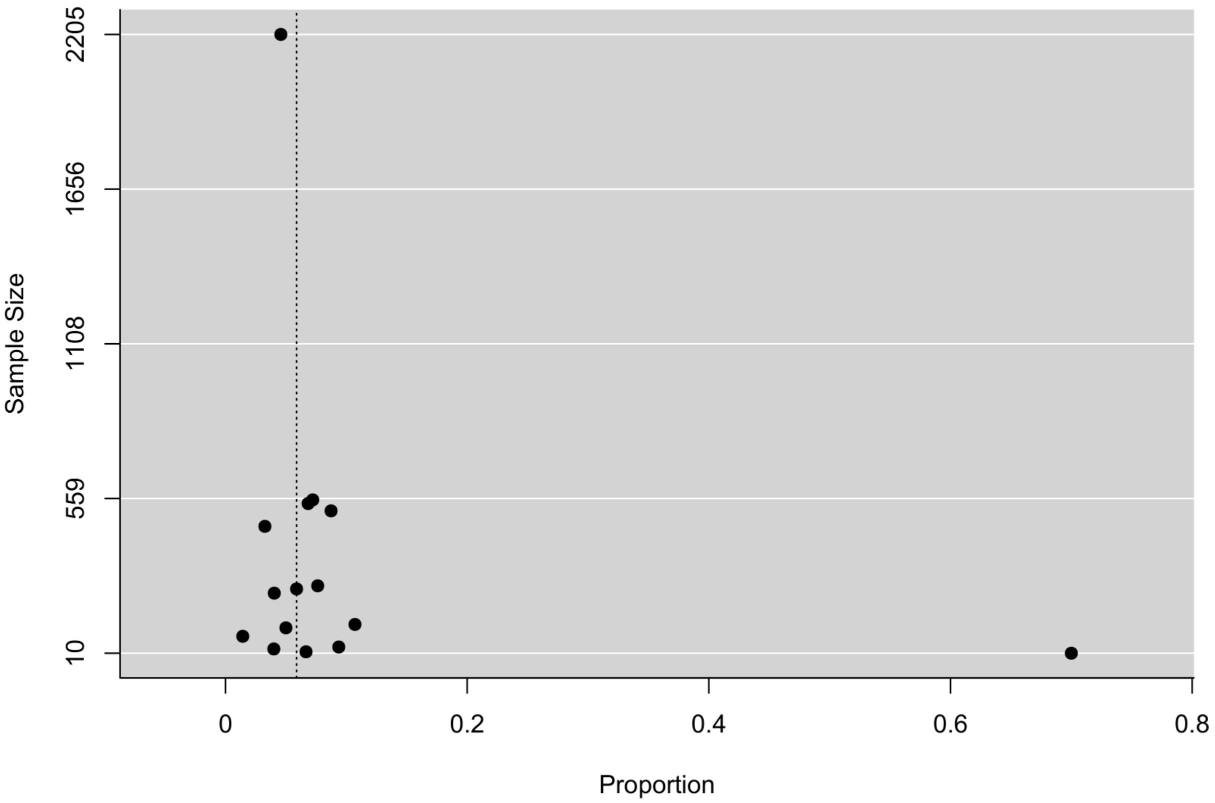

Figure S13. C5_TF_RIGHT_funnel sample size.

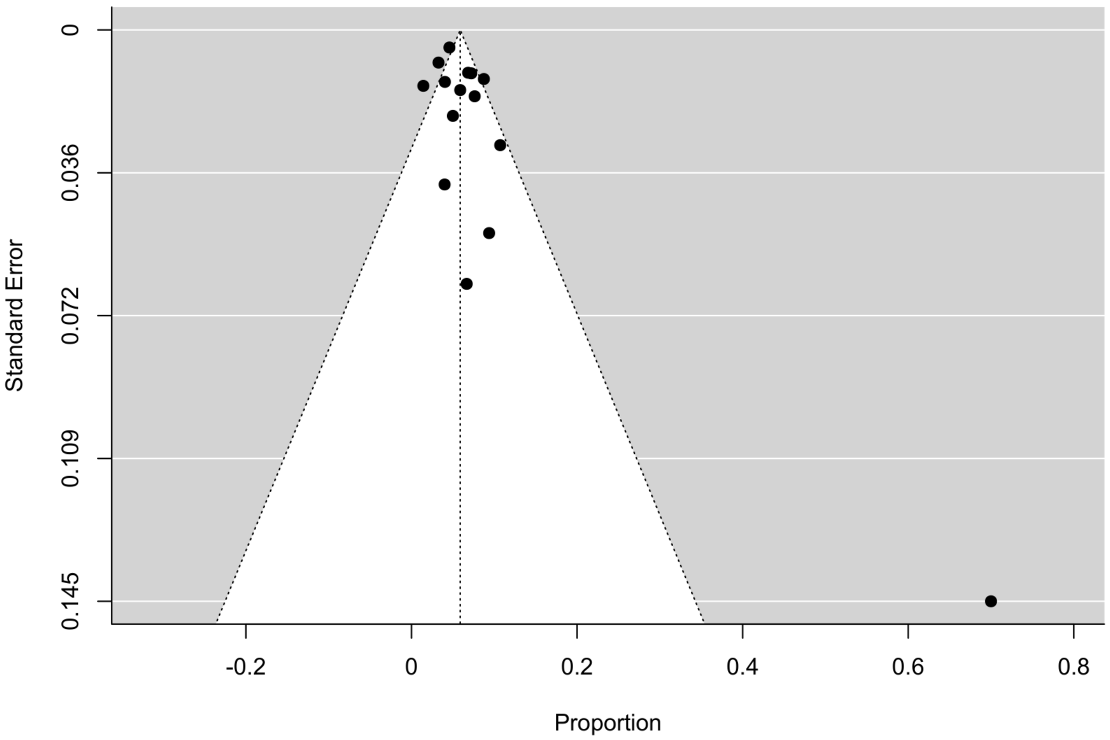

*

Figure S14. C5_TF_RIGHT_funnel SE.

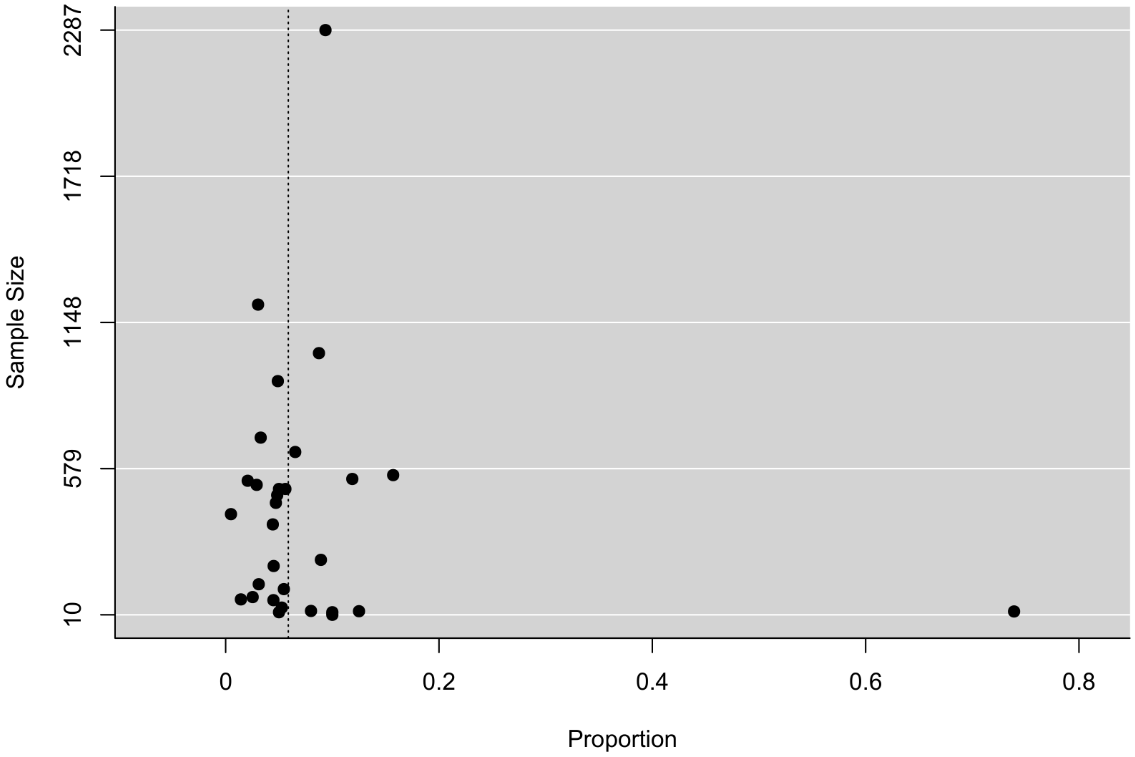

Figure S15. C5_TF_funnel sample size.

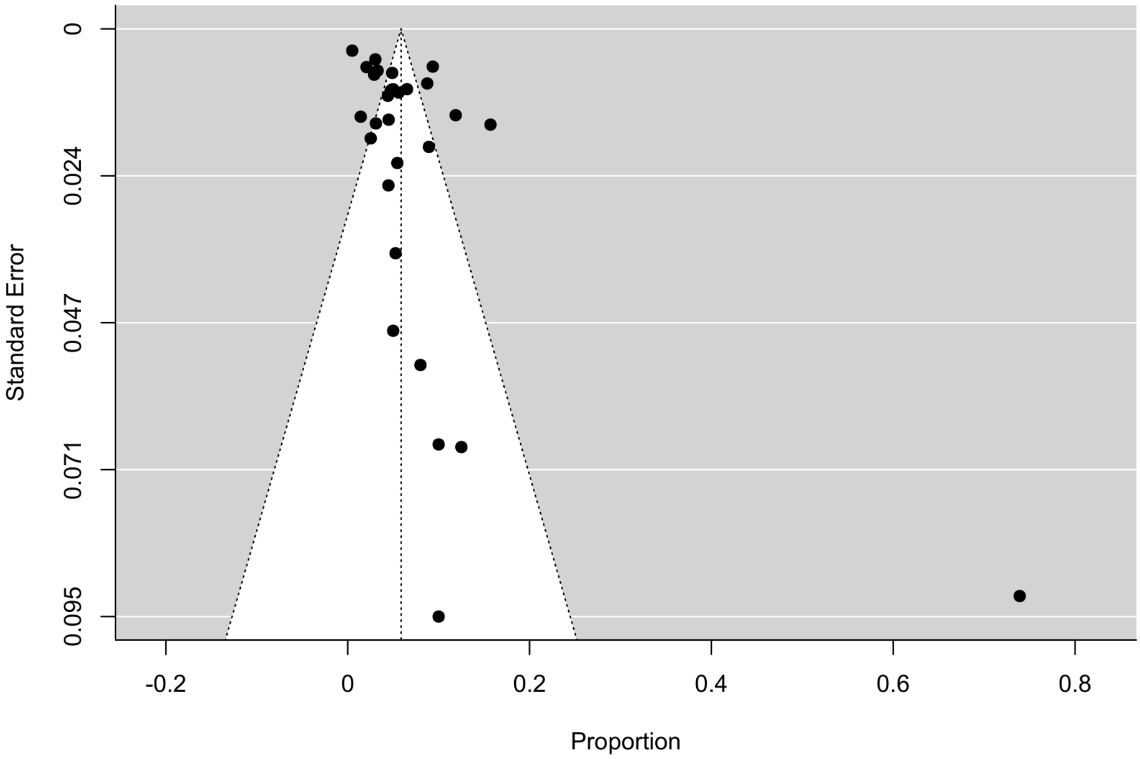

Figure S16. C5_TF_funnel SE.
